# Supplementary material for: Multi-isotopes in human hair: A tool to initiate cross-border collaboration in international cold-cases
Source: PLoS One. 2022 Oct 26;17(10):e0275902. doi: 10.1371/journal.pone.0275902 (PMC9603990; doi:10.1371/journal.pone.0275902)
Supplement: Attachment — Submitted filename: 2022-05-31 comments CL PONE-D-22-13353_edits.pdf [file pone.0275902.s010.pdf]

PLOS ONE

Multi-isotopes in Human Hair: A tool to initiate Cross-border Collaboration in International Cold-Cases

--Manuscript Draft--

**Manuscript Number:**	PONE-D-22-13353
**Article Type:**	Research Article
**Full Title:**	Multi-isotopes in Human Hair: A tool to initiate Cross-border Collaboration in International Cold-Cases
**Short Title:**	Multi-isotopes in Human Hair: A tool to initiate Cross-border Collaboration in International Cold-Cases
**Corresponding Author:**	Clement P. Bataille<br>University of Ottawa<br>Ottawa, Ontario CANADA
**Keywords:**	Stable isotope; Sulfur; Hydrogen; Hair; Cold-cases
**Abstract:**	Unidentified human remains have historically been investigated nationally by law enforcement authorities. However, this approach is outdated in a globalized world with rapid transportation means, where humans easily move long distances across borders. Cross-border cooperation in solving cold-cases is rare due to political, administrative or technical challenges. It is fundamental to develop new tools to provide rapid and cost-effective leads for international cooperation. In this work, we demonstrate that isotopes are effective screening tools to help identify cold-cases with potential international ramifications. We first complete an existing database of hydrogen and sulfur isotopes in hair of human residents across North America by compiling or analyzing hair from Canada, the United States and Mexico. Using these databases, we develop maps predicting isotope variations in human hair across the continent. We demonstrate that both hydrogen and sulfur isotopes in human hair are highly predictable and display strong spatial patterns. Multi-isotope analysis combined with dual hydrogen and sulfur isotope geographic probability maps provide evidence for international travel in two case studies. In the first, we demonstrate that multi-isotope analysis in hair of unidentified border crossers found in the United States, close to the Mexico-US border, help trace their origin back to specific regions of Mexico. These findings were validated by the subsequent identification through the Pima County Office of the Medical Examiner in Tucson, Arizona. In the second case study, we demonstrate that sequential multi-isotope analysis along the hair strands of an unidentified individual found in Canada provides detailed insights into the international mobility of this individual during the last year of life. In both cases, isotope data provide strong leads towards international travel.
**Order of Authors:**	Clement P. Bataille
	Saskia T.M. Ammer
	Shelina Bhuiyan
	Michelle M.G. Chartrand
	Gilles St-Jean
	Gabriel J. Bowen
**Additional Information:**	
**Question**	**Response**
**Financial Disclosure**<br><br>Enter a financial disclosure statement that describes the sources of funding for the work included in this submission. Review the submission guidelines for detailed	This project was supported by the Chemical, Biological, Radiological-Nuclear and Explosives Research and Technology Initiative [Award No. CRTI08-0116RD] awarded to G.S.J., the Canadian Security and Safety Program Targeted Investment [CSSP-2018-TI-2385] awarded to C.P.B. This research was further supported by the American Academy of Forensic Sciences (AAFS) Humanitarian and Human Rights Resource Center (HHRRC) grant, supported by the AAFS and National Institute of Justice (U.S.

Powered by Editorial Manager® and ProduXion Manager® from Aries Systems Corporation

requirements. View published research articles from *PLOS ONE* for specific examples.<br><br>This statement is required for submission and **will appear in the published article** if the submission is accepted. Please make sure it is accurate.<br><br>**Unfunded studies**<br>Enter: *The author(s) received no specific funding for this work.*<br><br>**Funded studies**<br>Enter a statement with the following details:<br>• Initials of the authors who received each award<br>• Grant numbers awarded to each author<br>• The full name of each funder<br>• URL of each funder website<br>• Did the sponsors or funders play any role in the study design, data collection and analysis, decision to publish, or preparation of the manuscript?<br>• **NO** - Include this sentence at the end of your statement: *The funders had no role in study design, data collection and analysis, decision to publish, or preparation of the manuscript.*<br>• **YES** - Specify the role(s) played.<br><br>* typeset	Department of Justice), awarded to S.T.M.A. Work by G.J.B. was supported by US National Science Foundation grants DBI-1565128 and DBI-1759730.
**Competing Interests**<br><br>Use the instructions below to enter a competing interest statement for this submission. On behalf of all authors, disclose any competing interests that could be perceived to bias this work—acknowledging all financial support and any other relevant financial or non-financial competing interests.<br><br>This statement is required for submission and **will appear in the published article** if the submission is accepted. Please make sure it is accurate and that any funding sources listed in your Funding Information later in the submission form are also declared in your Financial Disclosure	The authors have declared that no competing interests exist.

Powered by Editorial Manager® and ProduXion Manager® from Aries Systems Corporation

statement.<br><br>View published research articles from *PLOS ONE* for specific examples.<br><br>**NO authors have competing interests**<br><br>Enter: *The authors have declared that no competing interests exist.*<br><br>**Authors with competing interests**<br><br>Enter competing interest details beginning with this statement:<br><br>*I have read the journal's policy and the authors of this manuscript have the following competing interests: [insert competing interests here]*<br><br>* typeset	
**Ethics Statement**<br><br>Enter an ethics statement for this submission. This statement is required if the study involved:<br><br>• Human participants<br>• Human specimens or tissue<br>• Vertebrate animals or cephalopods<br>• Vertebrate embryos or tissues<br>• Field research<br><br>Write "N/A" if the submission does not require an ethics statement.<br><br>General guidance is provided below. Consult the submission guidelines for detailed instructions. **Make sure that all information entered here is included in the Methods section of the manuscript.**	The Office of Research Ethics and Integrity of the University of Ottawa approved this research program under protocol number [05-08-19]. Specifically, all sampling and analytical methods used to collect samples and information were in accordance with these regulations. Informed consent was obtained from all subjects or from their legal guardians in accordance with, and maintained under, IRB regulations.

**Format for specific study types**<br><br>**Human Subject Research (involving human participants and/or tissue)**<br>• Give the name of the institutional review board or ethics committee that approved the study<br>• Include the approval number and/or a statement indicating approval of this research<br>• Indicate the form of consent obtained (written/oral) or the reason that consent was not obtained (e.g. the data were analyzed anonymously)<br><br>**Animal Research (involving vertebrate animals, embryos or tissues)**<br>• Provide the name of the Institutional Animal Care and Use Committee (IACUC) or other relevant ethics board that reviewed the study protocol, and indicate whether they approved this research or granted a formal waiver of ethical approval<br>• Include an approval number if one was obtained<br>• If the study involved *non-human primates*, add *additional details* about animal welfare and steps taken to ameliorate suffering<br>• If anesthesia, euthanasia, or any kind of animal sacrifice is part of the study, include briefly which substances and/or methods were applied<br><br>**Field Research**<br><br>Include the following details if this study involves the collection of plant, animal, or other materials from a natural setting:<br>• Field permit number<br>• Name of the institution or relevant body that granted permission	
**Data Availability**<br><br>Authors are required to make all data underlying the findings described fully available, without restriction, and from the time of publication. PLOS allows rare exceptions to address legal and ethical concerns. See the PLOS Data Policy and FAQ for detailed information.	Yes - all data are fully available without restriction

Powered by Editorial Manager® and ProduXion Manager® from Aries Systems Corporation

A Data Availability Statement describing where the data can be found is required at submission. Your answers to this question constitute the Data Availability Statement and **will be published in the article**, if accepted.<br><br>**Important:** Stating 'data available on request from the author' is not sufficient. If your data are only available upon request, select 'No' for the first question and explain your exceptional situation in the text box.<br><br>Do the authors confirm that all data underlying the findings described in their manuscript are fully available without restriction?	
**Describe where the data may be found in full sentences. If you are copying our sample text, replace any instances of XXX with the appropriate details.**<br><br>• If the data are **held or will be held in a public repository**, include URLs, accession numbers or DOIs. If this information will only be available after acceptance, indicate this by ticking the box below. For example: *All XXX files are available from the XXX database (accession number(s) XXX, XXX.*).<br>• If the data are all contained **within the manuscript and/or Supporting Information files**, enter the following: *All relevant data are within the manuscript and its Supporting Information files.*<br>• If neither of these applies but you are able to provide **details of access elsewhere**, with or without limitations, please do so. For example:<br><br>*Data cannot be shared publicly because of [XXX]. Data are available from the XXX Institutional Data Access / Ethics Committee (contact via XXX) for researchers who meet the criteria for access to confidential data.*<br><br>*The data underlying the results presented in the study are available from (include the name of the third party*	All relevant data are within the manuscript and its Supporting Information files.

and contact information or URL).<br>• This text is appropriate if the data are owned by a third party and authors do not have permission to share the data.<br><br>* typeset	
Additional data availability information:	

Manuscript

Click here to access/download;Manuscript;SA_Manuscript_5522_CPB.docx

Multi-isotopes in Human Hair: A tool to initiate Cross-border Collaboration in International Cold-Cases

Clément P. Bataille[1*], Saskia T.M. Ammer[2,3], Shelina Bhuiyan[1], Michelle M.G. Chartrand[4], Gilles St-Jean[1], Gabriel J. Bowen[5]

[1]Department of Earth and Environmental Sciences, University of Ottawa, 120 University, Ottawa, ON Canada, K1N 6N5

[2] Geology & Geochemistry Cluster, Vrije Universiteit Amsterdam, De Boelelaan 1085, 1081 HV Amsterdam, the Netherlands

[3] Co van Ledden Hulsebosch Centre (CLHC), Science Park 904, 1098 XH, Amsterdam, the Netherlands

[4] Department of Earth and Environmental Science, University of Ottawa, 120 University, Ottawa, ON Canada, K1N 6N5; Current affiliation: National Research Council Canada, Metrology.

[5] Department of Geology & Geophysics and Global Change & Sustainability Center, University of Utah, Salt Lake City, UT, United States of America

* Correspondence to C.P.B. (email: cbataille@uottawa.ca

Abstract

Unidentified human remains have historically been investigated nationally by law enforcement authorities. However, this approach is outdated in a globalized world with rapid transportation means, where humans easily move long distances across borders. Cross-border cooperation in solving cold-cases is rare due to political, administrative or technical challenges. It is fundamental to develop new tools to provide rapid and cost-effective leads for international cooperation. In this work, we demonstrate that isotopes are effective screening tools to help identify cold-cases with potential international ramifications. We first complete an existing database of hydrogen and sulfur isotopes in hair of human residents across North America by compiling or analyzing hair from Canada, the United States and Mexico. Using these databases, we develop maps predicting isotope variations in human hair across the continent. We demonstrate that both hydrogen and sulfur isotopes in human hair are highly predictable and display strong spatial patterns. Multi-isotope analysis combined with dual hydrogen and sulfur isotope geographic probability maps provide evidence for international travel in two case studie[illegible]n the first, we demonstrate that multi-isotope analysis in hair of unidentified border crossers found in the United States, close to the Mexico-US border, help trace their origin back to specific regions of Mexico. These findings were validated by the subsequent identification through the Pima County Office of the Medical Examiner in Tucson, Arizona. In the second case study, we demonstrate that sequential multi-isotope analysis along the hair strands of an unidentified individual found in Canada provides detailed insights into the international mobility of this individual during the last year of life. In both cases, isotope data provide strong leads towards international travel.

Keywords: Stable isotope; Sulfur; Hydrogen; Hair; Cold-cases

1. Introduction

The establishment of Interpol many decades ago came from the necessity for international police cooperation in response to the rise of international crime organizations. Nevertheless, such international cooperation usually remains restricted to organized crime while significant barriers impede cooperation in the resolution of cold cases [1]. Countries lack a universal language, funding and the political will to harmonize law enforcement practices and facilitate cross-border cold case investigations [1]. A significant and increasing number of cold-cases are international, particularly in areas located close to land borders where remains of migrants or trafficking victims are common [2]. The remains of individuals with a foreign origin often stay unidentified due to the absence of documents, evidence and cooperation between law enforcement agencies [2]. When DNA or fingerprint databases are available (e.g., EU, North America), they strongly favor cross-border collaboration for those cases with potential international origin [3]. However, while DNA is considered the holy grail of identification, it is often inapplicable either because the DNA is too degraded for analysis, too costly or simply not useful because there is no known reference sample (*e.g.*, family reference sample or the decedent's DNA profile in a database) to compare the sample of the deceased individual to. It has been found that the more socially marginalized the family of the missing is, the more obstacles the family has to face to obtain information about the loved one's whereabouts and to submit data to the appropriate authorities [4]. The administrative, technical, and cultural barriers that limit cooperation between law enforcement agencies are unlikely to disappear in the short term. There is therefore an urgent need to develop tools that provide robust leads to facilitate cross-border implications in the resolution of cold-cases [5].

Stable isotopes are ubiquitous intrinsic markers with the potential to contribute new and actionable evidence in cold-cases, even those involving long-term unidentified individuals [6–13]. Isotopes compose all organic molecules and their abundances in human tissues inform about a person's diet, health, or mobility history providing critical information about human remains [6,8,14–19]. Isotope data from hair have been increasingly used in investigating cold-cases because hair is easily collected and resistant [20], and isotope data in hair is usually preserved post-mortem [21,22]. While bulk isotope data is often used in forensic cases, sequential isotope profiles along human hair can provide chronological information about the diet and location changes of an unidentified decedent at approximately monthly to bi-monthly resolutions as hair grows at ~7-10 mm/month, though not continuously [6,10,16,23–30]. Isotope data in hair could provide a key screening tool to identify those cold-cases that have a potential international outlook. However, the application of this isotope geolocation technology at the international scale requires the development of cross-border databases of isotope composition in hair and models predicting the isotope patterns in the tissue of interest.

Many isotopes have the potential to trace individual mobility. However, some (e.g., metals) are not directly integrated within the hair structure and are easily exchanged or contaminated by exogenous sources (e.g., bathing water, aerosols, post-mortem exposure) [19,22]. Consequently, when measured in hair, those isotopes reflect local contamination by soil and water at the site where human remains were found and are not useful for solving cold cases [31,32]. Hydrogen/oxygen and sulfur, however, compose keratin [33], are resistant to post—mortem exposure [34] and are thus the ideal candidate for multi-isotope geolocation of humans from their hair. The ^{2}H and ^{18}O abundances ($\delta^{2}H$ and $\delta^{18}O$ values respectively) in humans reflect primarily the isotopic variability in drinking water which follows systematic spatiotemporal patterns along latitude, elevation, and continentality [28]. When an individual moves through the landscape, its hair inherits the isotopic composition of the drinking water consumed along the way and can thus be used to reconstruct origin and movements [32]. Though limited in the geographic resolution of the information it can provide, $\delta^{2}H$ in human hair has been used for decades in isotope geolocation studies [6,10,16,24–30] and data is available from many countries to develop continental-scale maps of isotope variations (or isoscapes) [35].

The S isotope composition ($\delta^{34}S$) in human hair is inherited from the isotopic composition of the food consumed with little isotopic fractionation [36]. Plants and crops, at the base of food systems, uptake S from two main sources: S-containing bedrock minerals or inorganic fertilizers, which generally have low but variable $\delta^{34}S$, and marine aerosols which have high $\delta^{34}S$ values [37]. The mixing of these two isotopically distinct S sources controls a large part of $\delta^{34}S$ variations observed in ecosystems and ancient human societies with high $\delta^{34}S$ values in coastal environments and progressively lower $\delta^{34}S$ moving inland [37–39]. In modern times, however, the supermarket human diet mixes products from multiple distant locations and sources, complicating the interpretation of $\delta^{34}S$ values [36,40–43]. But even in modern societies, the $\delta^{34}S$ values in resident human hair display clear differences between countries and continents [36,40–43]. For example, hair of North American residents have distinctively lower $\delta^{34}S$ values than those of Asians or Europeans [36,40–43]. Bataille et al. (2020) further demonstrated that, within a country, systematic and predictable $\delta^{34}S$ gradients were present [41]. The predicted $\delta^{34}S$ values in hair of Canadian residents could unambiguously distinguish “true” residents from “snowbirds” traveling to the tropics to escape the Canadian winter.

Carbon and nitrogen isotope compositions ($\delta^{13}C$ and $\delta^{15}N$) in human hair are also composing keratin and resistant to post-mortem exposure [34]. Both carbon and nitrogen come primarily

from diet but can, in certain cases, bring some geolocation information [44]. $\delta^{13}C$ values in human hair primarily track dietary habits of an individual, particularly the proportion of C_4 vs. C_3 plant-derived products consumed [20,21,44]. This is because C_3 crops including beet, barley, rice, potato, or wheat have in average much lower $\delta^{13}C$ values (~-25‰) [45] than C_4 plants including corn, soybean, millet, and cane sugar (~-12‰) [45]. However, some $\delta^{13}C$ trends, independent of personal dietary choices, also exist at the regional up to the global scales due to the distinct mix of C_4 vs. C_3 products in food systems [11]. For example, in North America, more C_3 crops (e.g., wheat, barley, beats) are cultivated in the North and dry continental interiors whereas more C_4 crops (e.g., corn, sugar cane) are cultivated in the South and coastal regions. This spatial organization of food systems leads to distinct $\delta^{13}C$ values in human hair transmitted through the preferential consumption of local food [41,42,46]. Similar spatial patterns exist for the $\delta^{15}N$ in hair at the regional to global scale due to differences in agricultural practises [11]. However, $\delta^{15}N$ values in human hair are mostly controlled by dietary choices with more positive $\delta^{15}N$ values for individuals eating more seafood in coastal regions [47].

The main objective of this study is to develop a framework to use multi-isotope geolocation from modern human hair to assist in solving international cold cases. We first analyze and/or compile δ^2H and $\delta^{34}S$ values from hair of residents of Canada, the United States and Mexico. We use these databases to generate maps predicting δ^2H and $\delta^{34}S$ values in hair across North America. We then analyze the δ^2H, $\delta^{34}S$, $\delta^{13}C$ and $\delta^{15}N$ values in bulk hair of undocumented border-crossers (UBCs) that died at the Mexico-US border and were later identified through the Pima County Office of the Medical Examiner. We compare the predicted geographic origins from bulk hair dual H and S isotope analysis with their known-origin. We finally run a sequential analysis of δ^2H, $\delta^{34}S$, $\delta^{13}C$ and $\delta^{15}N$ values along the length of a Canadian cold case. We assess the potential mobility of the individual and the possibility of cross-border traveling.

2. Materials and Methods

2.1 Ethics statement

The Office of Research Ethics and Integrity of the University of Ottawa approved this research program under protocol number [05-08-19]. Specifically, all sampling and analytical methods used to collect samples and information were in accordance with these regulations. Informed consent was obtained from all subjects or from their legal guardians in accordance with, and maintained under, IRB regulations.

2.2 Hair samples

2.2.1 Residents' hair samples from across North America:

We analyzed or compiled 692 $\delta^{34}S$ (Database_S1) and 846 δ^2H (Database_S2) from hair of North American residents. Out of these samples 649 sites have both δ^2H and $\delta^{34}S$ data (Database_S3). We obtained 101 Mexican residents' hair samples collected by Dr. Ammer in 2019 following protocols described in [42]. These samples were analysed for δ^2H values at the Jan Veizer Stable Isotope Laboratory. These samples had already been analyzed for $\delta^{34}S$ values [42]. We compiled 210 δ^2H and 60 $\delta^{34}S$ data from American residents' hair samples analyzed in [28] and [36], respectively. The data generated by Ehleringer et al. (2008) were analyzed using older protocols and calibration standards. We transformed the hair δ^2H values using the function *refTrans* in the assignR package to place them on the same calibration scale as the rest of the hair data described in [35]. We obtained 592 Canadian residents' hair samples collected by Dr. Chartrand between 2007 and 2012. 531 of these samples were

previously analyzed for δ^{34}S values [41]. 535 samples were analyzed for δ^2H in 2013 though the results are only available in a non-peer-reviewed report [48]. The data generated by Chartrand were analyzed using older protocols and calibration standards. We transformed the hair δ^2H values using the function *refTrans* in the assignR package to place them on the same calibration scale as the rest of the hair data as described in [35].

2.2.2 United States – Mexico undocumented border crosser hair samples

The remains of undocumented border crossers are often found by border patrol, non-governmental institutions or private citizens in the rural regions along the Mexico-US border. Remains found throughout most of southern Arizona are then transported to the Pima County Office of the Medical Examiner (PCOME). As part of Dr. Ammer's doctoral thesis, hair samples from four undocumented border crossers were collected by pulling the hair with the roots (Table 1). These individuals were found relatively rapidly after their death (<5 weeks) limiting potential effect of decomposition on the isotope values [34]. Additionally, the individuals have been tentatively identified by authorities through various means and await final confirmation. Those identifications can be used, with caution, as a mean to validate isotope-based geographic assignments. All hair samples were approximately 4 cm long, thus representing the last few months of these individuals' lives. We analyzed the bulk hair for δ^2H, δ^{13}C, δ^{15}N and δ^{34}S (Table 2 and Data_S1). It is assumed that the bulk analysis of isotope in hair of those UBCs, by and large, reflect the region of last residence. However, the journey to the border can sometimes take up to months and in rare cases, even years as some reside close to the border awaiting their chance to cross. Further, some of the individuals might have crossed the border multiple times after living in the US and being deported. Based on the investigative work and contact with the potential family of the deceased, it is thought that UBC#2 lived in the US for a few months and was subsequently deported and died during his subsequent crossing back into the US. The metadata on the four undocumented border crossers can be found in Table 1.

Table 1. Metadata on the four undocumented border crossers.

UBC #	Sex, Age at Death	Country, State, City of Origin	Border Patrol Corridor Found	Surface Management	Latitude/Longitude of recovery	Body condition	Post Mortem Interval
UBC #1	Male, 28	Mexico, Sinaloa, Jitzamuri	Goldwater	Cabeza Prieta National Wildlife Refuge	32.3475;-113.3066 (precise to within ca. 300ft./100m)	Decomposed w/ focal skeletonization	< 3 weeks
UBC #2	Male, 61	Mexico, Tlaxcala, Tlacatecpa	San Miguel	Tohono Oodham Nation	31.8997;-111.769884 (precise to within ca. 300ft./100m)	Decomposed w/ focal skeletonization	< 3 weeks
UBC #3	Male, 27	Mexico, Guerrero, Ayulta de los Libres	San Miguel	Tohono Oodham Nation	31.890533;-112.163068 (precise to within ca. 300ft./100m)	Decomposed w/ focal skeletonization	< 3 weeks
UBC #4	Male, 20	Guatemala, Huehuetenang, San Ildefonso	San Miguel	Tohono Oodham Nation	31.735367/-111.835983 (precise to within ca. 300ft./100m)	Skeletonization w/ mummification	< 5 weeks

2.2.3 Canadian cold-case

In 2008, the RCMP Halifax Detachment sent a clump of dreadlock hair to the University of Ottawa from an unidentified individual recovered near the Halifax airport (hereafter called Mr. Halifax; RCMP Case File #2004-3757)). The remains of this individual were found on October 8, 2004 near the Halifax city airport, Nova Scotia, Canada. A procedure developed and tested in a previous study to align, combine and sequence multiple hair was used [32]. However, the hair of this individual was extremely brittle which made alignment challenging. Consequently, individual locks of hair were used as the basis for alignment, combination and segmentation. We prepared a total of 27, 0.5 cm samples, each representing about half a

month timeframe. Due to the difficulty in aligning the dreadlocks, the time uncertainty and averaging represented by the isotope time-series are probably higher than in previous studies causing, in this case, a more "elastic" timeframe than usual [31,32]. We analyzed each segment for δ^2H, δ^{13}C, δ^{15}N and δ^{34}S (Data_S2).

2.3 Isotope analysis

The Mexican resident bulk hair, Mexico-US border migrant bulk hair and Canadian cold-case hair segments were all analyzed following the same procedure at the Jan Veizer Stable Isotope Laboratory at the University of Ottawa, Canada. Hair was first washed in a series of three baths of 2:1 solution of chloroform:methanol ($CHCl_3$:MeOH). The hair was then dried, ground using a Retsch ball mill and stored in glass vials until analyzed.

For δ^{13}C and δ^{15}N analysis, the hair segment samples and isotope standards (approximately 400 μg each) were weighed into tin capsules and loaded onto an Elemental Analyser (Vario EL III, Elementar, Germany) interfaced with a ConFlo III (Thermo, Germany) to an isotope ratio mass spectrometer (IRMS, Delta^{Plus} Advantage, Thermo, Germany). One tin capsule was analyzed for each sample. Internal standards used for calibration were a mix of ammonium sulfate and sucrose ($\delta^{13}C_{VPDB}$, −11.94‰; $\delta^{15}N_{AIR}$, 16.58‰), nicotinamide ($\delta^{13}C_{VPDB}$, −22.95‰; $\delta^{15}N_{AIR}$, 0.07‰), and caffeine ($\delta^{13}C_{VPDB}$, −28.53‰; $\delta^{15}N_{AIR}$, −3.98‰). All δ^{15}N and δ^{13}C values are reported versus AIR and V-PDB, respectively. Two USGS human hair standards, USGS 42 (δ^{13}C= -21.09 ± 0.10 ‰; δ^{15}N= 8.05 ± 0.10 ‰) and USGS 43 (δ^{13}C= -21.28 ± 0.10‰; δ^{15}N= 8.44 ± 0.10‰), were used for quality check. Analytical precision is based on an internal QC sample (glutamic acid, which is not used for normalization) and is usually better than ±0.2‰ for both δ^{13}C and δ^{15}N.

The δ^2H of the non-exchangeable hydrogen of hair was determined using the comparative analysis approach described by Wassenaar and Hobson [49] based on two USGS calibrated keratin hydrogen-isotope reference materials (CBS: −157‰, KHS: −35.3‰). We performed hydrogen isotopic measurements on H_2 gas derived from high-temperature (1400 °C) flash pyrolysis (TCEA, Thermo) of 150 ± 10 ug hair subsamples and keratin standards loaded into silver capsules. Resultant separated H_2 was analyzed on an interfaced Thermo Delta V Plus continuous-flow isotope-ratio mass spectrometer (Bremen, Germany). All δ^2H values are reported to the international scale VSMOW-SLAP. Measurement of the two keratin laboratory reference materials corrected for linear instrumental drift were both accurate and precise with typical within-run measurement error < 2‰. Two USGS human hair standards, USGS 42 (δ^2H= -72.9 ± 2.2 ‰) and USGS 43 (δ^2H= -44.4 ± 2.0 ‰), were used as quality checks. Analytical precision is based on the reproducibility of the USGS hair standards is better than ± 2‰.

For δ^{34}S analyses, the samples and standards were weighed into tin capsules, loaded onto an Elementar Isotope Cube Elemental Analyser, and flash combusted at 1800°C. The EA method was optimized for SO_2, where N_2 and CO_2 were not retained. SO_2 was trapped and released to the Conflo IV (Thermo, Germany) interfaced to the IRMS (DeltaPlus XP, with a special 6 collector sulfur cups array (SO-SO_2), Thermo, Germany). Standards used for calibration were silver sulphides: IAEA-S-1 (δ^{34}S= -0.3‰), IAEA-S-2 (δ^{34}S= 22.7‰), and an internal standard S-6 (δ^{34}S= -0.7‰). All δ^{34}S values were reported to the international scale VCDT. Two USGS human hair standards, USGS 42 (δ^{34}S= 7.84 ± 0.25 ‰) and USGS 43 (δ^{34}S=10.46 ± 0.22 ‰) were used as quality checks. Analytical precision is based on the reproducibility of the USGS hair standards is better than ± 0.3‰.

2.4 Isoscapes

Geographic probability maps compare the observed isotopic value for a biological tissue (e.g., hair) with that predicted by an isoscape [50].

2.4.1 Sulfur hair isoscape

Bataille et al. (2020) predicted the $\delta^{34}S$ trends of modern human hair across Canada demonstrating a strong gradient from coast to more inland provinces reflecting the influence of local food systems [41]. Similar trends are observed in the $\delta^{34}S$ in hair of residents of the United States [36] and, to a lesser extent, Mexico [42]. Based on these observations, we used an existing framework to generate a $\delta^{34}S$ isoscape from hair of North American residents [38,41]. We assembled data on selected covariates that represent the main factors that impact variability in $\delta^{34}S$ values, including country of residence (Canada, USA or Mexico), geology (age), climate (e.g., precipitation, temperature), soil proprieties (e.g., pH, clay content, organic matter), aerosol deposition (e.g., sea salt) and distance to the coast. We resampled and re-projected all the selected environmental geospatial products into WGS84-Eckert IV 1km^2 resolution and used the latitude and longitude of each sampling site to extract the local values for each raster. We combined the $\delta^{34}S$ hair compilation and the extracted covariate values at each site into a regression matrix and used generalized linear model (GLM) regression kriging to predict $\delta^{34}S$ in hair across North America using the "caret" package [51]. We first use the Akaike information criterion (AIC) to estimate prediction error and rank the relative quality of GLM models. We then used simple kriging to map the remaining variance of the residuals (Script_S1).

2.4.2 Hydrogen hair isoscape

We generated a δ^2H isoscape for human hair following the procedure described in the assignR package [52]. The process requires a tissue-specific isotope dataset of known-origin individuals to develop a transfer function between an isoscape and the tissue of interest. We used the compiled dataset of δ^2H in hair combining the δ^2H in hair of Canadian and Mexican residents analyzed in this study with published δ^2H in hair of US residents and incorporated this dataset to the known origin sample database in the assignR package. We calibrated the hair δ^2H isoscape using the *calRaster* function in the assignR package.

2.5 Probability maps

We used the metrics generated by the *QA* function in assignR to compare the quality of probability maps generated from each isoscape. We then used the continuous-surface probability framework from the assignR package [52] (i.e., *pdRaster* function) to estimate the most likely locations of origin of each individual from the bulk hair single (δ^2H or $\delta^{34}S$) and dual (δ^2H and $\delta^{34}S$) isotope data from Mexican-US border crossers and from each segment of the Canadian cold-case (Script_S2).

3. Results

3.1 Sulfur isoscape

The 692 compiled and analysed $\delta^{34}S$ data from hair of North American residents are normally distributed (Shapiro Test, p-value<0.05). $\delta^{34}S$ values range from -1.4‰ to 8‰ and average 2.3‰. Some geographical regions of North America are underrepresented in the dataset: the eastern USA, western USA and southern USA.

The best GLM model (AIC=556) used latitude, longitude, country of origin, and distance to the coast as the dominant predictors of the $\delta^{34}S$ values (Fig 1A). This GLM explained 66% of the variance with a Root Mean Square Error (RMSE) of 0.79‰ (Fig 1A). After kriging the

residuals using a Gaussian variogram model (Fig. 1B), the resulting regression kriging model explains 77% of the variance with a RMSE of 0.65 ‰ (Fig 1C). The value of 0.65‰ represents ~8% of the full range of measured $\delta^{34}S$ values over the dataset. Latitude and country of origin are the strongest predictor of $\delta^{34}S$ values. Going north to south, Canadian samples, on average, have lower $\delta^{34}S$ values than Americans and Mexicans. Distance to the coast and longitude are also key predictors with individuals living close to the coast having higher $\delta^{34}S$ values than those living more inland.

The regression kriging produced a $\delta^{34}S$ isoscape in human hair that displays strong spatial patterns associated with country and distance to the coast. Sites located in coastal North America have higher $\delta^{34}S$ values (Fig. 1D). The highest values are found in western coastal Mexico. Conversely, sites located in interior regions of Canada have the lowest $\delta^{34}S$ values.

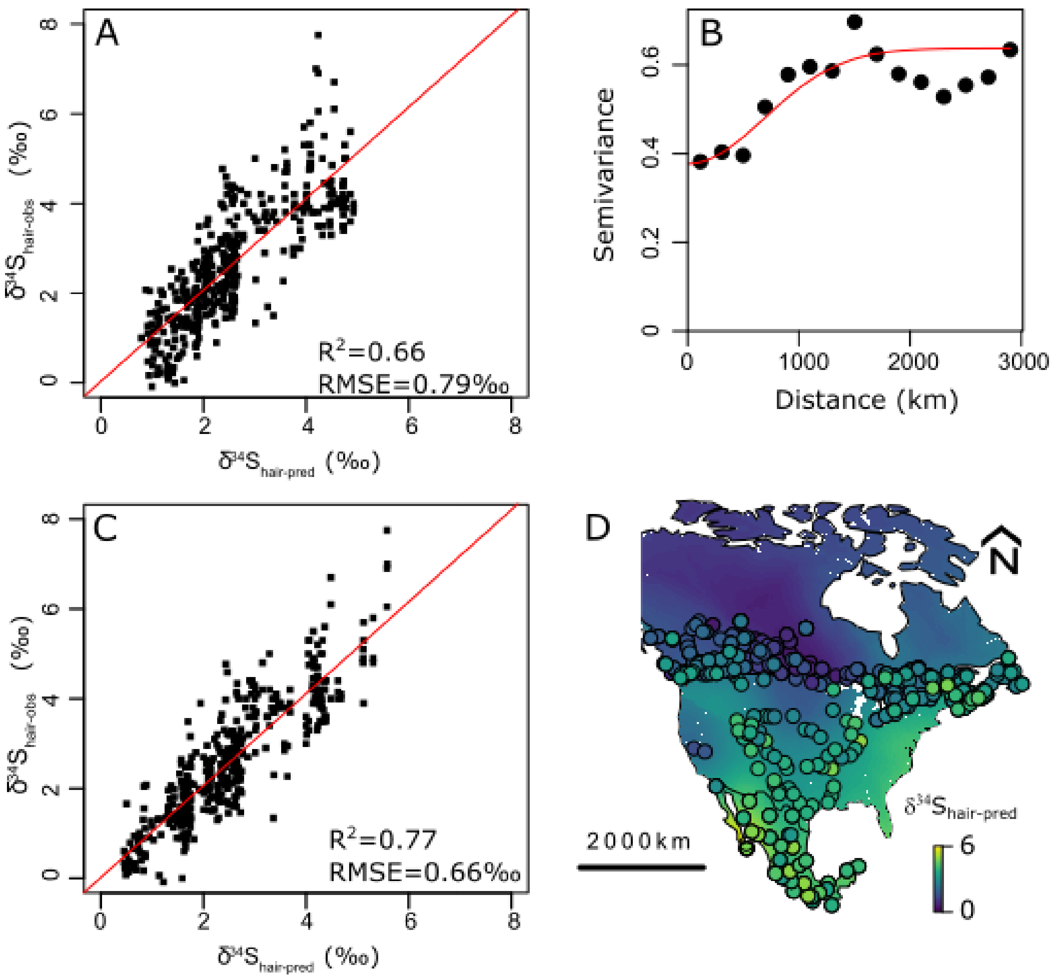

Figure 1. Sulfur isoscape generated by regression kriging. A: Cross-validation between measured and predicted $\delta^{34}S$ values using a Generalized Linear Model (GLM) regression using distance to the coast, longitude, latitude and country as predictors. RMSE = Root Mean Square Error. The red line is the best-fit linear model. **B:** Variogram of the GLM regression residuals. Red line is a Gaussian model fit using simple kriging. **C.** Cross-validation between measured and predicted $\delta^{34}S$ values using GLM regression kriging. RMSE = Root Mean Square Error. The red line is the best-fit linear model. **D.** Map of $\delta^{34}S$ in hair of resident humans across North America with locations of collection sites from residents (including individuals from this study as well as published data). The R script to generate these figures is available in **R_Script_1**.

3.2 Hydrogen isoscape

The 846 compiled and analysed δ^2H data from hair of North American residents are normally distributed (Shapiro Test, p-value<0.05). δ^2H values range from -119.8‰ to -37.4‰ and average -79‰. We observed a strong correlation between δ^2H values in hair and δ^2H values in precipitation (Fig. 2A). The produced δ^2H isoscape in human hair displays strong spatial patterns (Fig. 2B).

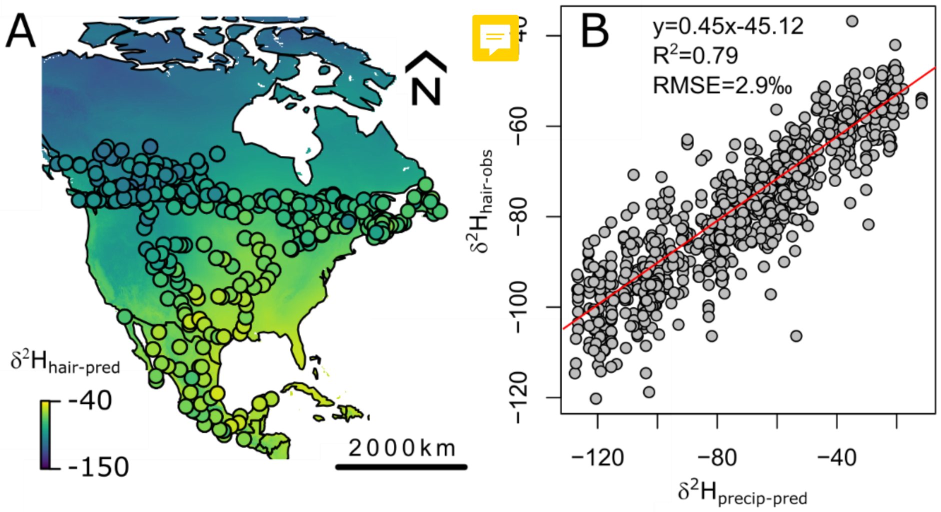

Figure 2. Hydrogen isoscape generated in the assignR package. A: Map of δ^2H in hair of residents from North America with locations of collection sites from residents (including individuals from this study as well as published data) and **B:** Calibration equation between modeled δ^2H in precipitation and hair from North American residents (including individuals from this study as well as published data).

3.3 Isotope-based probability map results

3.3.1 Quality evaluation of isotope-based probability maps

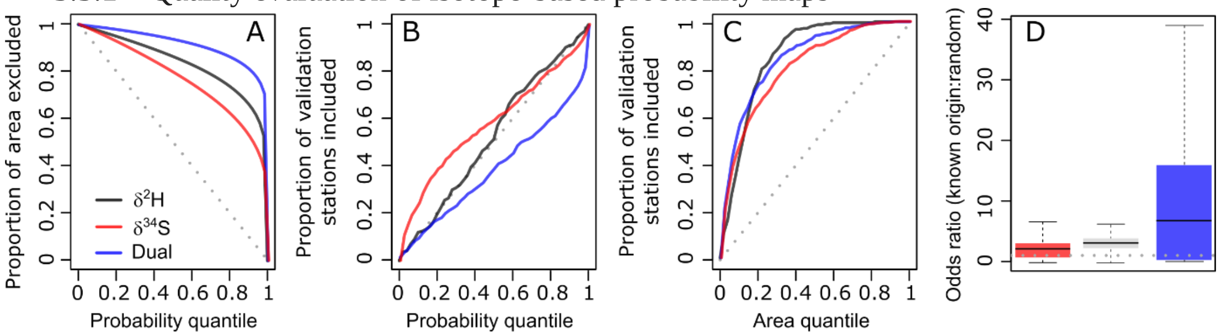

Figure 3. Quality evaluation of hydrogen, sulfur and dual-isotope probability maps. These plots were generated using the *QA* function in the assignR package. **A.** Proportion of the study area excluded from the assignments as a function of probability threshold. Higher values indicate a potential for more specific assignments. **B.** Proportion of validation samples correctly assigned as a function of the probability threshold. If accurate posterior probabilities are estimated for each sample, these values should fall along the 1:1 line. **C.** Proportion of validation samples correctly assigned as a function of the area quantile, providing an integrated measure of assignment sensitivity. **D.** The distribution of odds ratios for the known origins of the validation samples relative to random quantifies the strength of isotopic support for one location relative to another. Higher odds ratios indicate more specific assignments.

Quality metrics for the single- and dual-isotope analyses suggest strong potential for this method to provide accurate and specific information on the origin of human hair samples, and highlight the added power of the dual-isotope method (Fig. 3). The area-exclusion plot (Fig. 3A) shows that the spatial distribution of posterior probabilities is very uneven, particularly for the dual-isotope analysis. This implies that the isotopic information strongly discriminates between more- and less-likely regions and could be used to eliminate large parts of North America as a potential origin for a sample at a high level of certainty. The validation plot (Fig. 3B) shows that, for hydrogen isotopes, the proportion of samples correctly assigned to origin mostly scales as expected with the probability threshold adopted for assignment, implying that this isoscape and its uncertainty appropriately represent the human hair data. The validation plots show slight deviation from the expected proportion of samples correctly assigned for δ^{34}S and for dual isotopes (Fig. 3B). For δ^{34}S the proportion of stations included are overestimated relative to the probability quantile likely indicating that the modeled uncertainty is lower than represented in the isoscape. Surprisingly, however, the dual isotope show the opposite trend with the proportion of validation stations underestimated relative to the probability quantile indicating that the model misses the true origin of known-origin individuals more than expected (Fig. 3B). This could reflect some bias in the sulfur isotope predictions associated with the regression kriging approach and/or the validation approach. This bias would be accounted for in the univariate uncertainty but leads to incorrect predictions in the multivariate case. With larger dataset with more complete spatial coverage, it might be useful to test new modeling approach to predict δ^{34}S variations (e.g., random forest regression). The assignment power plot (Fig. 3C) uses the known origin data to test the ability of the method to correctly assign sample origin across a range of exclusion area thresholds. It shows that both single- and dual-isotope analyses have high power, though the δ^{2}H and dual-isotope method give substantial increase in power across all area thresholds relative to δ^{34}S. Finally, the odds ratios for the known locations of sample origin are substantially higher than the random value for all methods, but are approximately 5 time greater for the dual-isotope method than either single-isotope analysis (Fig. 3D). Collectively, these results support the validity of the isoscapes as a template for interpreting human hair isotope data and suggest that assignments made using the isotopic data, particularly in the case of dual-isotope analysis, can provide accurate and specific information on the geographic origin of samples.

3.3.2 US-Mexico border crossers cold cases results

The hair samples of the undocumented border crossers were bulk-analyzed, thus not segmented. Table 2 presents the δ^{2}H, δ^{34}S, δ^{13}C and δ^{15}N isotope values for each of the four individuals. The values are the average of three measurements. The δ^{13}C and δ^{15}N isotope values of the four UBCs were compared to the values previously published for residents of North America [36,41,42].

Based on the available δ^{15}N data from North America, an origin from the United States can be excluded for UBC #1, all other individuals fall within the ranges reported for all three countries (Table 2). The δ^{13}C values reported for two of the UBCs (UBC #3 and UBC #4) would exclude both the United States and Canada as potential countries of origin (Table 2). The δ^{13}C values for UBC #1 and UBC #2 fall within the higher end of the range of values found in the United States and Canada but would not allow for a clear distinction. The δ^{34}S values of UBCs #2 and #4 fall within the ranges reported for all three countries, while #1 and #3’s values are too high to have originated from Canada (Table 2).

The δ^{2}H values of all UBCs fall within the ranges reported for Mexico and the United States but are not compatible with Canada (Table 2).

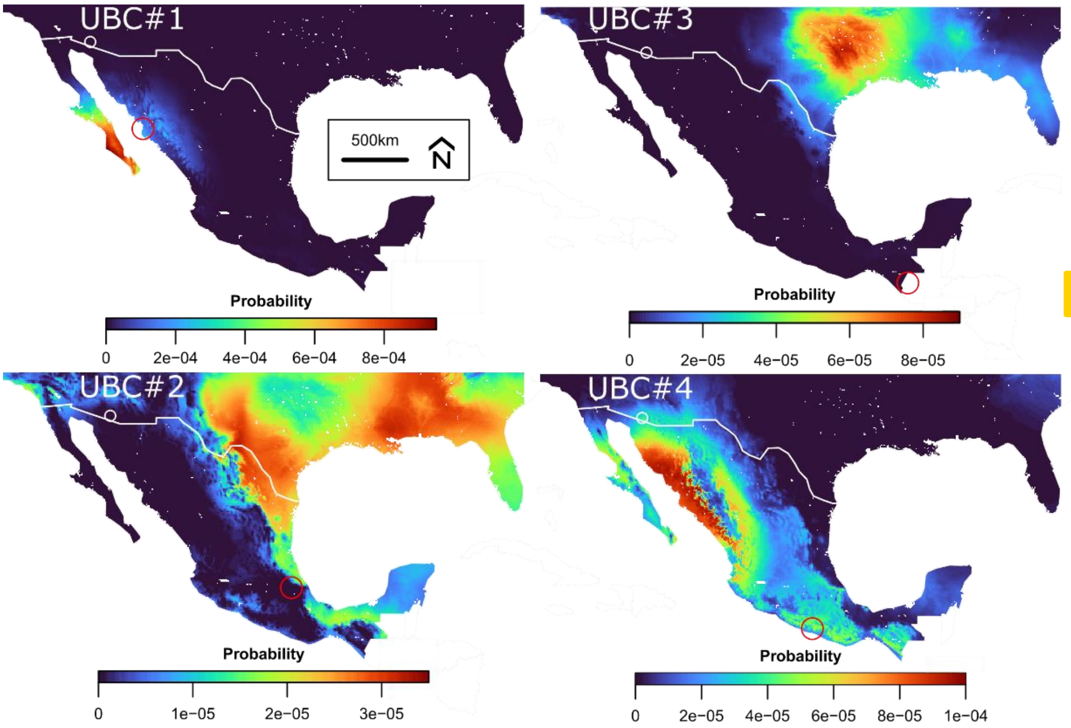

Figure 4 Probabilistic maps of provenance for Mexico-USA border crossers estimated from δ^2H and $\delta^{34}S$ values for bulk hair. Locations where the human remains were discovered, are indicated by white circles. Locations where the remains are thought to have originated, based in independent evidence, are marked with red circles. Colours on the maps depict the predicted probability of origin based on isotopic evidence.

We generated single and dual isotopes probabilistic maps of provenance for each individual and compared them to the location of origin tentatively identified by authorities (Fig. 4). UBC #1 probably originated from Jitzamuri, Sinaloa, Mexico. Only a few coastal regions of western Mexico are compatible with the isotope data from this individual's hair. Among those, the region around Jitzamuri shows high probability. UBC #2 probably originates from Tlacatecpa, Tlaxcala, Mexico. The dual isotopes probabilistic maps show low probability of origin in the purported region of origin except for some coastal regions along the Gulf of Mexico. Probable regions of origin include most of the southeastern USA, and coastal region around the Gulf of Mexico. UBC #3 probably originates from Ayulta de los Libres, Guerrero, Mexico. The dual isotope probabilistic maps show a high probability from this city but are not very specific as all the western coast of Mexico shows a high probability of origin with highest probability in northwestern Mexico. Most of northern Mexico and the southern USA regions are also compatible with the isotope values of this individual. UBC #4 most likely originated from San Ildefonso, Huehuetenango, Guatemala. Unfortunately, our map does not extend to Guatemala. The most probable regions of potential origin within our study areas is the south-central USA along the Mississippi river valley.

Table 2. Isotope results of the four undocumented border crossers. All data in ‰.

UBC #	δ^2H (VSMOW)	$\delta^{34}S$ (VCDT)	$\delta^{13}C$ (VPDB)	$\delta^{15}N$ (Air)
UBC #1	-51.7	6.2	-15.2	10.4
UBC #2	-57.4	3.6	-15.2	8.5
UBC #3	-51.6	5.0	-13.6	9.5
UBC #4	-57.3	3.2	-13.6	9.1
Mr. Halifax mean ± 1sd	-60.4 ± 2.6	2.9 ± 0.8	-19.1 ± 0.5	9.1 ± 0.2

	range	-57.2 to -69.3	0.9 to 4.2	-19.9 to -18.0	8.6 to 9.4
Canada	mean ± 1sd	-86.3 ± 12.6	1.7 ± 1	-18.5 ± 0.6	9.2 ± 0.5
	range	-60.2 to -120.5	-1.4 to 4.8	-20.3 to -16.7	7.6 to 10.8
USA	mean ± 1sd	-69.9 ± 13.2	3.4±1.1	-17.2±0.8	8.9±0.4
	range	-37.2 to -106.2	0.9 to 5.0	-21.6 to -14.7	6.5 to 9.7
Mexico	mean ± 1sd	-59.2 ± 6	4.5±1.1	-15.6 ± 1	9.2±0.6
	range	-42 to -74	2.7 to 8.0	-18.3 to -12.8	6.8 to 10.8

3.3.3 Halifax cold case results

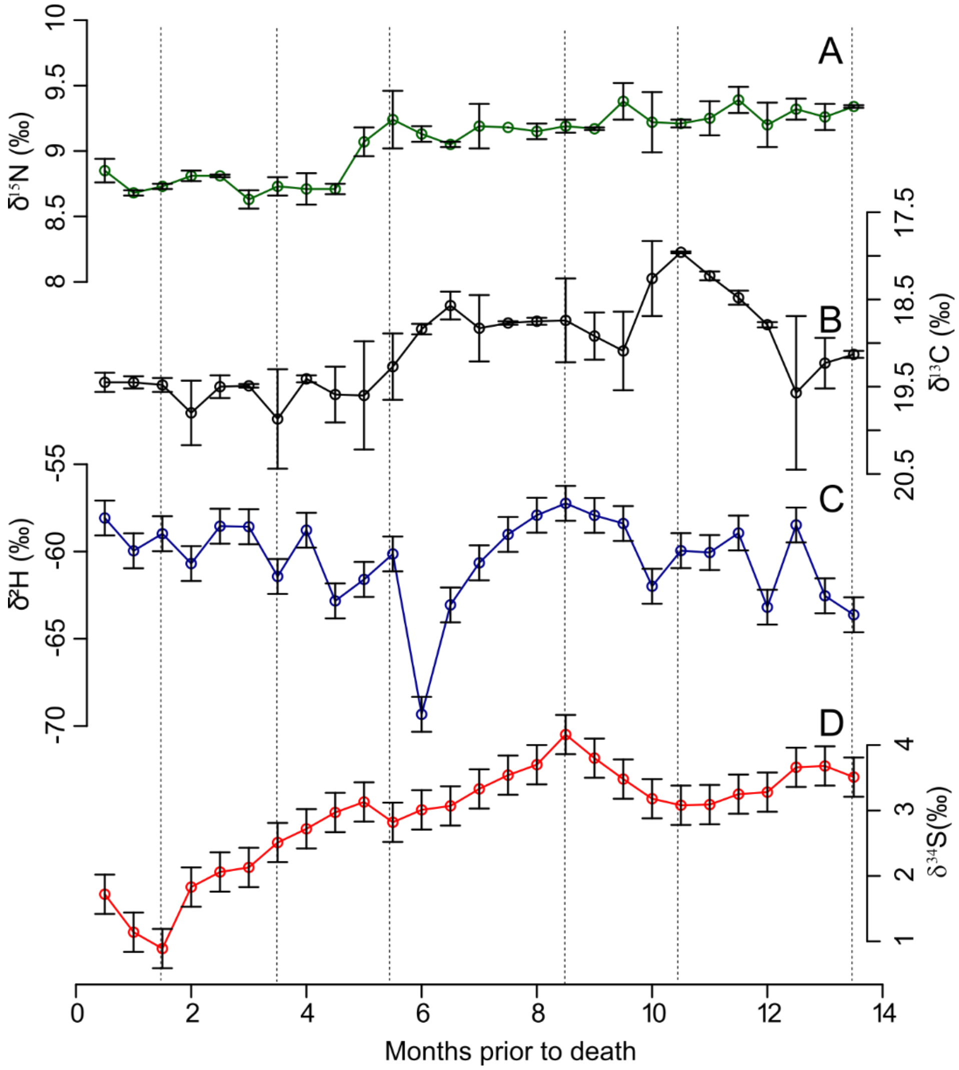

Figure 5 Isotope values in Mr. Halifax's hair profile (Complete data per hair segment available in the supplemental SI_Data). The length of the hair is converted into months prior to death (PTD) assuming a constant growth rate of 1cm per month. **A.** δ^{15}N values; **B.** δ^{13}C values; **C.** δ^{2}H values; **D.** δ^{34}S values. Dashed lines define isotope lines used for dual isotope probability maps in figure 6.

We compare the average δ^{13}C (-19.1 ± 0.5 ‰) and δ^{15}N (9.1 ± 0.3 ‰) values of Mr. Halifax's with previously published δ^{13}C and δ^{15}N values of North American residents [36,41,42] (Table 2). The δ^{15}N values are similar to the δ^{15}N values for Canada, the USA and Mexico. The profile shows two main zones of stable δ^{15}N values with values around 9.3 ‰ between 5 and 14 months PTD and a shift to lower values 5 months PTD (Fig. 5A). The average hair δ^{13}C value from Mr. Halifax is most similar to the average bulk hair δ^{13}C values of Canada's western and central provinces including western Ontario, Manitoba, and the Prairies.

However, the $\delta^{13}C$ values are higher between 5-12 months PTD and would be compatible with eastern Canada or the eastern USA during this interval (Fig. 5B). The $\delta^{2}H$ values of Mr. Halifax's hair ranged between -57.2 and -69.3‰. Compared to the $\delta^{2}H$ values of North American residents, Mr. Halifax's hair $\delta^{2}H$ value is most similar to the $\delta^{2}H$ values of hair from the eastern Canadian provinces, including Ontario, Quebec, the Maritimes, and Newfoundland (Table 2). Except for one very low value at 5 months PTD, the $\delta^{2}H$ values from Mr. Halifax's hair become more positive between 5-12 months PTD (Fig. 5C).

We used these isotope values to generate single and dual-isotope probabilistic geographic maps from each segment of hair. We summarized in Fig. 6 the map results by showing 6 individual segments corresponding to dotted lines in figure 5. Dual $\delta^{2}H$ and $\delta^{34}S$ values from the base of the hair (1.5 cm, 3.5 cm and 5.5 cm) are compatible with the Canadian urban centers around the Great Lakes from Winnipeg to Sudbury and with US Midwest cities along the Mississippi river valley. At 8.5 months PTD, dual-isotope values are compatible with the southeastern USA including Florida but are not compatible with regions in Canada. At 13.5 months PTD, the dual-isotope values are compatible with the Canadian Maritimes including Nova Scotia and Newfoundland but also with a broad region in the southeastern and southern USA.

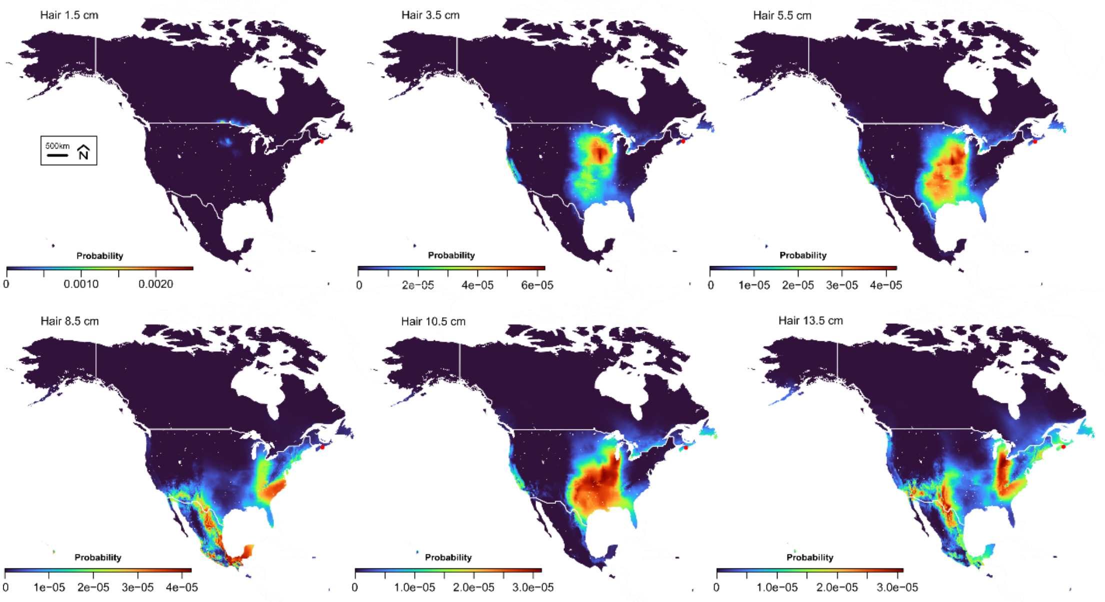

Figure 6 Isotope-based probabilistic maps of provenance for Mr. Halifax estimated from $\delta^{2}H$ and $\delta^{34}S$ values for different representative hair segments. Locations where the human remains were discovered (Halifax, NS) are indicated by red points. Colours on the maps depict the predicted probability of origin.

4. Discussion

4.1 Isocapes

Since the seminal work of Ehleringer et al. (2008), country-scale studies analyzing isotopes in human hair of residents and/or tap water have become increasingly available providing the basis to develop isoscapes in many regions of the world [28,41–43,53–56]. Efforts to harmonize isotope analyses have also facilitated the integration of data generated from different laboratories, countries and times [35].

4.1.1 Sulfur isoscape

As observed in previous studies [36,40,41,43], the $\delta^{34}S$ values in human hair are distinct for different countries across North America. $\delta^{34}S$ values in Canada average 1.7±1.0 ‰, $\delta^{34}S$ values in the United States average 3.4±1.1 ‰ and $\delta^{34}S$ values in Mexico average 4.4±1.0 ‰. Different countries have different food production systems, supply chains and dietary habits that give them distinct baseline $\delta^{34}S$ values [41]. Within countries, particularly Canada and the USA, we observe a trend between $\delta^{34}S_{hair}$ values and distance to the coast. $\delta^{34}S$ in North American crops should reflect the mixture of isotopically light sulfates from the soil solution, and isotopically heavy marine aerosols [36,41,43,57,58]. As food systems become more distant from the coast, bedrock S or anthropogenic sources dominate decreasing $\delta^{34}S$ values. The $\delta^{34}S_{hair}$ variability in North American residents closely follows this isotopic pattern in food systems likely because customers obtain a large part of their S from locally sourced high-protein food items (e.g., meat, yogurt, cheese, eggs). Even though North American residents have a supermarket diet and homogenized dietary habits, the regional patterns in $\delta^{34}S$ in ecosystems are transmitted to human hair through the importance of regional food supply in sulfur intake. Eating locally has become a more powerful movement in the last decade where locally produced foods have become more available and prized. The difference in sea salt aerosol deposition between North American countries might also explain their distinct average hair $\delta^{34}S$ values and the trend in $\delta^{34}S$ values with latitude. Mexico receives more marine sulfate than the United States and Canada because populations are on average closer to the coast in Mexico. The $\delta^{34}S$ isoscape developed from this regression kriging approach shows excellent predictive power and strong spatial patterns that are promising for identifying cross-border cold cases.

4.1.2 Hydrogen isoscape

As observed in previous studies [28,53,59], the $\delta^{2}H$ values in human hair follow a continuous gradient across North America (Fig. 2A). When $\delta^{2}H$ hair data is corrected to be on the same reference scale [35], we can explain 80% of the variations in $\delta^{2}H$ hair across North America using only the isotopic signal from modeled precipitation (Fig. 2B). This strong relationship between local water and hair $\delta^{2}H$ values reflects the local nature of water sources in human societies [28]. Drinking water has usually local to regional origin depending on the source [54–56]. Similarly, most beverages are derived from local to regional water sources including bottled water, coffee, tea or even soft drinks and milk [60]. Some of the residuals between precipitation and $\delta^{2}H$ values in hair probably derive from differences between the $\delta^{2}H$ of local precipitation and that of the consumed tap water. Tap water from different sources at one location might have different isotopic compositions. For example, tap water from shallow groundwater usually reflects an average of local precipitation $\delta^{2}H$ [61] whereas tap water from deep aquifers, lakes or river waters might have more distant or fossil sources (e.g., glacial water) and/or might have been evaporated [54,61]. This isotopic difference between water sources is exacerbated at the continental scale because different countries and regions rely on different water sources. For example, in North America, Mexico supplies the majority of its tap water from groundwater sources whereas Canada and the USA use primarily surface water sources. Differences in dietary choices, tap water sources and beverage inputs between participants living in a single location can also contribute to distinct hair $\delta^{2}H$ values at a single location [60]. Many other factors can influence the relationship between precipitation $\delta^{2}H$ and $\delta^{2}H$ in human hair including the consumption of imported drinks with non-local $\delta^{2}H$ values, the presence of non-local study participants or uncertainties in the predicted precipitation $\delta^{2}H$ values. Despite those limitations, the continental $\delta^{2}H$ isoscape shows excellent predictive power and strong spatial patterns that are promising for identifying cross-border cold cases.

4.2 Interest in multi-isotope provenancing in international forensic studies

Stable isotopes have shown promise for provenancing unidentified decedents from cold-cases investigated by local jurisdictions [6,31,62–64] or to identify remains recovered from the sites of past wars and conflicts [7,38,39,62,65]. However, the development of international isotope baselines from modern residents offers new possibilities to use isotopes for contemporary cross-border forensic applications [7,8,65]. In our globalized world, many forensic questions have international components. Through two case studies, we show that using dual S and H isotope provenancing provides critical leads to facilitate international forensic collaborations.

Lack of documents, fingerprint, dental, medical records and family members to obtain family reference samples or antemortem samples for DNA comparison makes it very challenging to identify UBCs at the US-Mexico border. These difficulties are further enhanced by the inability to narrow down the search area to more probable options. The four individuals studied here were recently tentatively identified and serve as a basis to test the use of dual-isotope provenancing for identification purposes. Out of the 4 UBC studied, none showed a local origin in the western US. All the individuals have dual-isotope values that place their origin in more southern regions of North America. As we only analysed isotope values in bulk hair for these individuals, we were not expecting precise provenancing because the isotope data in hair could reflect a mixture of isotope values from their last location of reference with isotope values inherited during their journey to the border. Specifically, the durations of travel can vary greatly, from merely days to up to months, and in rare cases even years. This largely depends on the availability of funds, previous extortions through cartels, previous (negative) experiences, place of origin, health status and, among many other variables personal decision-making. Even with this temporal resolution limitation, the dual-isotope data show promising results. The individuals UBC #1 and UBC #4 show high isotope-based probabilities in the city/region of inferred origin of the UBC. UBC #2 likely resided for several months in the U.S. before being deported, re-entering and perishing in the US, explaining the strong signal from the southern USA. The origin of UBC #3 could not be properly inferred because our predictions did not include Guatemala. Analyzing isotope data sequentially along the hair and from other tissues (e.g., teeth and bones) could thus substantially help reconstruct a more detailed travel history for those individuals.

While Mr. Halifax was found near an international airport, the forensic inquiry around this cold-case has remained national. The dual S and H isotope provenancing reveal that this individual traveled across large distances in the year prior to his death (PTD). During the few months PTD, the individual lived either somewhere in eastern Canada, very likely in a municipality along the shore of the Great Lakes such as Sudbury, Sault-Saint-Marie or Winnipeg or in the northern US Midwest region. However, earlier in his life (i.e., 5 to 14 months PTD), the dual-isotope provenancing indicate travel and/or residence in the southeastern USA. During the 5 to 12 months PTD interval, some probability maps of provenance are incompatible with any region in Canada. Dual isotope probability maps between 12 to 14 months PTD show some potential Canadian origin in coastal Nova Scotia or Newfoundland but are mostly compatible with the southeastern USA. The carbon isotope data further support the proposed dual-isotope geographic assignments. While it is challenging to use carbon isotope for producing probabilistic maps of provenance because of fractionation associated with individual physiology and diet, the $\delta^{13}C$ trend within a hair profile does record mobility [32]. In the case of Mr. Halifax, carbon isotope trends strongly validate the dual S and H isotope geographic assignments. Extremely low $\delta^{13}C$ values (~ -19 ‰), a few months PTD, are compatible with regions of Ontario around the Great Lakes (i.e., western Ontario and Manitoba). However, between 5 to 14 months PTD, a shift towards higher $\delta^{13}C$ values culminating at -18 ‰ is more typical of the USA. Even the $\delta^{15}N$ values, which are usually not

sensitive to mobility within a given country [32], appear to record a shift from lower to higher $\delta^{15}N$ values around 5 months PTD. The higher $\delta^{15}N$ values a year PTD possibly reflect a shift in dietary habits between places of residence (e.g., more fish consumption in the southeastern USA leading to higher $\delta^{15}N$ values).

This study demonstrates the potential of using multi-isotope analyses, and isotope-based geographic assignments as a rapid response tool in international cold-cases. The power of isotope provenancing increases several folds when multiple isotope systems are combined. The precision of dual-isotope results varies, but in some cases, the potential areas of origin that are identified are very specific. Reconstructing the movement history and origin with isotope data provides critical leads to law enforcement agencies for cross-border collaborations. The case of UBC #4 demonstrates the importance of large-scale and continuous isoscapes that are as spatially extensive as possible. This not only applies to undocumented border crossers at the US-Mexico border but also to any other case of unidentified human remains with a potentially international outlook. Incomplete isoscapes bear the risk of exclusion or inclusion of regions that may otherwise be ex/included. This can severely affect the investigative work and thus slow down the identification process.

5. Conclusion

In this work, we demonstrated that developing continental-scale isoscapes for sulfur and hydrogen in hair is feasible and yields accurate models with strong predictive potential for isotope-based provenancing. As more isotope data from human tissues are published and integrated, those models will become increasingly accurate and useful for provenance. We then show how those isoscapes are particularly useful to provide evidence for cross-border mobility in unidentified individuals. Through a first case study, we show that probabilistic maps of provenance derived from bulk isotope analysis of UBC at the US-Mexico border help identify their country/region of origin. Through a second case study, we show that sequential multi-isotope analysis along a hair strand combined with continuous probabilistic maps of provenance yields a detailed travel history of an unidentified individual between eastern Canada and the southeastern USA. This type of evidence indicating international travel is essential to engage collaborations between law enforcement agencies. Now that the isotope baselines are established, we argue that multi-isotope profiling in human hair combined with isotope-based probabilistic provenancing provide a rapid, practical, inexpensive and powerful tool to screen cold-cases for potential international leads.

Acknowledgments

This project was supported by the Chemical, Biological, Radiological-Nuclear and Explosives Research and Technology Initiative [Award No. CRTI08-0116RD] awarded to G.S.J., the Canadian Security and Safety Program Targeted Investment [CSSP-2018-TI-2385] awarded to C.P.B. This research was further supported by the American Academy of Forensic Sciences (AAFS) Humanitarian and Human Rights Resource Center (HHRRC) grant, supported by the AAFS and National Institute of Justice (U.S. Department of Justice), awarded to S.T.M.A. Work by G.J.B. was supported by US National Science Foundation grants DBI-1565128 and DBI-1759730. The authors would like to thank the anonymous hair donors for supporting this research. The authors would also like to thank Paul Middlestead and the U of Ottawa's Jan Veizer Stable Isotope Laboratory for assistance in stable isotope analysis.

Author information

Contributions

Conceived and designed the experiments: C.P.B., S.A.. Performed the experiments: M.M.G.C. and S.B. Analyzed the data: C.P.B., S.A., G.J.B., M.M.G.C. G.S.J and S.B.. Wrote the paper: C.P.B., S.A.

Competing Interests

The authors declare no competing interests.

Corresponding author

Correspondence to Clement P. Bataille.

References

1. Barnett M, Coleman L. Designing police: Interpol and the study of change in international organizations. Int Stud Q. 2005;49. doi:10.1111/j.1468-2478.2005.00380.x

2. Haynes DF. Used, abused, arrested and deported: Extending immigration benefits to protect the victims of trafficking and to secure the prosecution of traffickers. Hum Rights Q. 2004;26. doi:10.1353/hrq.2004.0021

3. Toom V, Granja R, Ludwig A. The Prüm Decisions as an Aspirational regime: Reviewing a Decade of Cross-Border Exchange and Comparison of Forensic DNA Data. Forensic Sci Int Genet. 2019;41. doi:10.1016/j.fsigen.2019.03.023

4. Reineke RC. Naming the Dead: Identification and Ambiguity Along the U.S.-Mexico Border. The University of Arizona. 2016.

5. Goad G. Expanding Humanitarian Forensic Action: An Approach to U.S. Cold Cases. Forensic Anthropol. 2020;3. doi:10.5744/fa.2020.1006

6. Meier-Augenstein W, Fraser I. Forensic isotope analysis leads to identification of a mutilated murder victim. Sci Justice. 2008;48: 153–159. doi:10.1016/j.scijus.2007.10.010

7. Chesson LA, Meier- Augenstein W, Berg GE, Bataille CP, Bartelink EJ, Richards MP. Basic principles of stable isotope analysis in humanitarian forensic science. Forensic Science and Humanitarian Action. 2020. doi:10.1002/9781119482062.ch20

8. Kramer RT, Bartelink EJ, Herrmann NP, Bataille CP, Spradley K. Application of stable isotopes and geostatistics to infer region of geographical origin for deceased

undocumented Latin American migrants. Forensic Science and Humanitarian Action. 2020. doi:10.1002/9781119482062.ch27

9. Lehn C, Kalbhenn EM, Rossmann A, Graw M. Revealing details of stays abroad by sequential stable isotope analyses along human hair strands. Int J Legal Med. 2019;133: 935–947. doi:10.1007/s00414-018-1866-9

10. Lehn C, Rossmann A, Graw M. Provenancing of unidentified corpses by stable isotope techniques - presentation of case studies. Sci Justice. 2015;55: 72–88. doi:10.1016/j.scijus.2014.10.006

11. Hülsemann F, Lehn C, Schneider S, Jackson G, Hill S, Rossmann A, et al. Global spatial distributions of nitrogen and carbon stable isotope ratios of modern human hair. Rapid Commun Mass Spectrom. 2015;29: 2111–2121. doi:10.1002/rcm.7370

12. Meier- Augenstein W. Forensic stable isotope signatures: Comparing, geo- locating, detecting linkage. Wiley Interdiscip Rev Forensic Sci. 2019;1: e1339. doi:10.1002/wfs2.1339

13. Bartelink EJ, Chesson LA. Recent applications of isotope analysis to forensic anthropology. Forensic Sci Res. 2019;4: 29–44. doi:10.1080/20961790.2018.1549527

14. Bol R, Marsh J, Heaton THE. Multiple stable isotope (^{18}O, ^{13}C, ^{15}N and ^{24}S) analysis of human hair to identify the recent migrants to a rural community in SW England. Rapid Commun Mass Spectrom. 2007;21: 2951–2954. doi:10.1002/rcm.3168

15. Emery M V., Prowse TL, Elford S, Schwarcz HP, Brickley M. Geographic origins of a War of 1812 skeletal sample integrating oxygen and strontium isotopes with GIS-based multi-criteria evaluation analysis. J Archaeol Sci Reports. Elsevier; 2017;14: 323–331. doi:10.1016/J.JASREP.2017.06.007

16. Font L, Van Der Peijl G, Van Leuwen C, Van Wetten I, Davies GR. Identification of the geographical place of origin of an unidentified individual by multi-isotope analysis. Sci Justice. 2015;55: 34–42. doi:10.1016/j.scijus.2014.06.011

17. Vautour G, Poirier A, Widory D. Tracking mobility using human hair: What can we learn from lead and strontium isotopes? Sci Justice. Elsevier; 2015;55: 63–71. doi:10.1016/j.scijus.2014.10.001

18. Font L, Jonker G, Van Aalderen PA, Schiltmans EF, Davies GR. Provenancing of unidentified World War II casualties: Application of strontium and oxygen isotope analysis in tooth enamel. Sci Justice. 2015;55: 10–17. doi:10.1016/j.scijus.2014.02.005

19. Rodiouchkina K, Rodushkin I, Goderis S, Vanhaecke F. Longitudinal isotope ratio variations in human hair and nails. Sci Total Environ. 2022;808.

doi:10.1016/j.scitotenv.2021.152059

20. Petzke KJ, Boeing H, Metges CC. Choice of dietary protein of vegetarians and omnivores is reflected in their hair protein 13C and 15N abundance. Rapid Communications in Mass Spectrometry. 2005. pp. 1392–1400. doi:10.1002/rcm.1925

21. Macko SA, Engel MH, Andrusevich V, Lubec G, O'Connell TC, Hedges REMM. Documenting the diet in ancient human populations through stable isotope analysis of hair. Philos Trans R Soc B Biol Sci. 1999;354: 65–76. doi:10.1098/rstb.1999.0360

22. Kootker LM, von Holstein ICC, Broeders J, Wescott DJ, Davies GR, Mickleburgh HL. The effects of decomposition and environment on antemortem H-Pb-Sr isotope compositions and degradation of human scalp hair: Actualistic taphonomic observations. Forensic Sci Int. 2020;312. doi:10.1016/j.forsciint.2020.110336

23. Remien CH, Adler FR, Chesson LA, Valenzuela LO, Ehleringer JR, Cerling TE. Deconvolution of isotope signals from bundles of multiple hairs. Oecologia. 2014;175: 781–789. doi:10.1007/s00442-014-2945-3

24. Chau TH, Tipple BJ, Hu L, Fernandez DP, Cerling TE, Ehleringer JR, et al. Reconstruction of travel history using coupled δ^{18}O and ^{87}Sr/^{86}Sr measurements of hair. Rapid Commun Mass Spectrom. 2017;31: 583–589. doi:10.1002/rcm.7822

25. Fraser I, Meier-Augenstein W, Kalin RM. Stable isotope analysis of human hair and nail samples: The effects of storage on samples. J Forensic Sci. 2008;53: 95–99. doi:10.1111/j.1556-4029.2007.00607.x

26. Lugli F, Cipriani A, Tavaglione V, Traversari M, Benazzi S. Transhumance pastoralism of Roccapelago (Modena, Italy) early-modern individuals: Inferences from Sr isotopes of hair strands. Am J Phys Anthropol. 2018;167: 470–483. doi:10.1002/ajpa.23643

27. Codron J, Kirkman K, Duffy KJ, Sponheimer M, Lee-thorp JA, Ganswindt A, et al. free-ranging African elephants (Loxodonta africana) reveal dietary niche differences within populations. Can J Zool. 2013;91: 124–134. doi:10.1139/cjz-2012-0155

28. Ehleringer JR, Bowen GJ, Chesson LA, West AG, Podlesak DW, Cerling TE. Hydrogen and oxygen isotope ratios in human hair are related to geography. Proc Natl Acad Sci. National Academy of Sciences; 2008;105: 2788–2793. doi:10.1073/pnas.0712228105

29. Meier-Augenstein W, Kemp HF. Stable Isotope Analysis: Hair and Nails. In: Jamieson A, Moenssens A, editors. Wiley Encyclopedia of Forensic Science. Chichester, UK: John Wiley & Sons, Ltd; 2012.

30. Meier-Augenstein W. Stable Isotope Forensics: Methods and Forensic Applications of Stable Isotope Analysis. Developments in forensic science. Chichester, UK: John Wiley & Sons, Ltd; 2018.

31. Fauberteau AE, Chartrand MMG, Hu L, St-Jean G, Bataille CP. Investigating a cold case using high-resolution multi-isotope profiles in human hair. Forensic Chem. 2021;22. doi:10.1016/j.forc.2020.100300

32. Hu L, Chartrand MMG, St-Jean G, Lopes M, Bataille CP. Assessing the Reliability of Mobility Interpretation From a Multi-Isotope Hair Profile on a Traveling Individual. Front Ecol Evol. 2020;8: 302. doi:10.3389/fevo.2020.568943

33. Nehlich O. The application of sulphur isotope analyses in archaeological research: A review. Earth-Science Rev. 2015;142: 1–17. doi:10.1016/j.earscirev.2014.12.002

34. Saul TB, Chesson LA, Steadman DW, Gordon GW. Considerations for Stable Isotope Analysis of Human Hair: The Impact of Postmortem Environmental Exposure. Forensic Anthropol. 2021;4: 35. Available: https://link.gale.com/apps/doc/A689947949/AONE?u=anon~cbf1a205&sid=googleScholar&xid=df0bee2a

35. Magozzi S, Bataille CP, Hobson KA, Wunder MB, Howa JD, Contina A, et al. Calibration chain transformation improves the comparability of organic hydrogen and oxygen stable isotope data. Methods Ecol Evol. 2021; doi:10.1111/2041-210X.13556

36. Valenzuela LO, Chesson LA, O'Grady SP, Cerling TE, Ehleringer JR. Spatial distributions of carbon, nitrogen and sulfur isotope ratios in human hair across the central United States. Rapid Commun Mass Spectrom. 2011;25: 861–868. doi:10.1002/rcm.4934

37. Tcherkez G, Tea I. 32S/34S isotope fractionation in plant sulphur metabolism. New Phytol. 2013;200: 44–53. doi:10.1111/nph.12314

38. Bataille CP, Jaouen K, Milano S, Trost M, Steinbrenner S, Crubézy É, et al. Triple sulfur-oxygen-strontium isotopes probabilistic geographic assignment of archaeological remains using a novel sulfur isoscape of western Europe. PLoS One. Public Library of Science; 2021;16: e0250383. Available: https://doi.org/10.1371/journal.pone.0250383

39. Colleter R, Bataille CP, Dabernat H, Pichot D, Hamon P, Duchesne S, et al. The last battle of Anne of Brittany: Solving mass grave through an interdisciplinary approach (paleopathology, biological anthropology, history, multiple isotopes and radiocarbon dating). PLoS One. Public Library of Science; 2021;16: e0248086. Available: https://doi.org/10.1371/journal.pone.0248086

40. Valenzuela LO, Chesson LA, Bowen GJ, Cerling TE, Ehleringer JR. Dietary heterogeneity among western industrialized countries reflected in the stable isotope ratios of human hair. PLoS One. 2012;7: e34234. doi:10.1371/journal.pone.0034234

41. Bataille CP, Chartrand MMG, Raposo F, St-Jean G. Assessing geographic controls of hair isotopic variability in human populations: A case-study in Canada. PLoS One. 2020; doi:10.1371/journal.pone.0237105

42. Ammer S, Bartelink E, Vollner J, Anderson B, Cunha E. Socioeconomic and geographic implications from carbon, nitrogen, and sulfur isotope ratios in human hair from Mexico. Forensic Sci Int. 2020;316. doi:10.1016/j.forsciint.2020.110455

43. Lee HS, Shim JY, Shin WJ, Choi SH, Bong YS, Lee KS. Dietary homogenization and spatial distributions of carbon, nitrogen, and sulfur isotope ratios in human hair in South Korea. PLoS One. 2021;16. doi:10.1371/journal.pone.0256404

44. O'Brien DM. Stable Isotope Ratios as Biomarkers of Diet for Health Research. Annu Rev Nutr. 2015;35: 565–594. doi:10.1146/annurev-nutr-071714-034511

45. O'Leary MH. Carbon isotope fractionation in plants. Phytochemistry. 1981. doi:10.1016/0031-9422(81)85134-5

46. Tipple BJ, Valenzuela LO, Chau TH, Hu L, Bataille CP, Chesson LA, et al. Strontium isotope ratios of human hair from the United States: Patterns and aberrations. Rapid Commun Mass Spectrom. 2019;33. doi:10.1002/rcm.8378

47. Post DM. Using stable isotopes to estimate trophic position: Models, methods and assumptions. Ecology 2002 pp. 703–718. doi:10.1890/0012-9658(2002)083[0703:USITET]2.0.CO;2

48. Chartrand M, Gilles S-J. Forensic Attribution of CBRNE Materials: A Chemical Fingerprint Database Final Status Report Defence Research and Development Canada. Ottawa; 2015; 97. Available: http://cradpdf.drdc-rddc.gc.ca/PDFS/unc200/p801299_A1b.pdf

49. Wassenaar LI, Hobson KA, Sisti L. An online temperature-controlled vacuum-equilibration preparation system for the measurement of δ2H values of non-exchangeable-H and of δ18O values in organic materials by isotope-ratio mass spectrometry. Rapid Commun Mass Spectrom. 2015;29. doi:10.1002/rcm.7118

50. Wunder MB. Using isoscapes to model probability surfaces for determining geographic origins. Isoscapes: Understanding movement, pattern, and process on Earth through isotope mapping. Dordrecht: Springer Netherlands; 2010. pp. 251–270. doi:10.1007/978-90-481-3354-3_12

51. Kuhn M. Building Predictive Models in R Using the caret Package. J Stat Softw. 2008;28: 1–26. doi:10.1053/j.sodo.2009.03.002

52. Ma C, Bowen GJ. assignR: Infer Geographic Origin from Isotopic Data. 2019.

53. Gautam MK, Song BY, Shin WJ, Bong YS, Lee KS. Spatial variations in oxygen and hydrogen isotopes in waters and human hair across South Korea. Sci Total Environ. 2020;726. doi:10.1016/j.scitotenv.2020.138365

54. Wang S, Zhang M, Bowen GJ, Liu X, Du M, Chen F, et al. Water Source Signatures in the Spatial and Seasonal Isotope Variation of Chinese Tap Waters. Water Resour Res. 2018;54. doi:10.1029/2018WR023091

55. de Wet RF, West AG, Harris C. Seasonal variation in tap water δ2H and δ18O isotopes reveals two tap water worlds. Sci Rep. 2020;10. doi:10.1038/s41598-020-70317-2

56. Bowen GJ, Ehleringer JR, Chesson LA, Stange E, Cerling TE. Stable isotope ratios of tap water in the contiguous United States. Water Resour Res. 2007;43. doi:10.1029/2006WR005186

57. Stack P, Rock L. A δ34S isoscape of total sulphur in soils across Northern Ireland. Appl Geochemistry. 2011;26: 1478–1487. doi:10.1016/j.apgeochem.2011.05.021

58. Sparks JM, Crowley BE, Rutherford MG, Jaggernauth D. Coastal proximity, orientation, and precipitation amount drive spatial variability in δ34S values on the Caribbean island of Trinidad. Appl Geochemistry. Pergamon; 2019;108: 104372. doi:10.1016/j.apgeochem.2019.104372

59. O'Brien DM, Wooller MJ. Tracking human travel using stable oxygen and hydrogen isotope analyses of hair and urine. Rapid Commun Mass Spectrom. 2007;21: 2422–2430. doi:10.1002/rcm.3108

60. Chesson LA, Podlesak DW, Erkkila BR, Cerling TE, Ehleringer JR. Isotopic consequences of consumer food choice: Hydrogen and oxygen stable isotope ratios in foods from fast food restaurants versus supermarkets. Food Chem. 2010;119: 1250–1256. doi:10.1016/j.foodchem.2009.07.046

61. Bowen GJ, Guo JS, Allen ST. A 3-D groundwater isoscape of the contiguous USA for forensic and water resource science. PLoS One. 2022;17: e0261651.

62. Bartelink EJ, Berg GE, Beasley MM, Chesson LA. Application of stable isotope forensics for predicting region of origin of human remains from past wars and conflicts. Ann Anthropol Pract. 2014;38: 124–136. doi:10.1111/napa.12047

63. Ehleringer JR, Thompson AH, Podlesak DW, Bowen GJ, Chesson LA, Cerling TE, et al. A framework for the incorporation of isotopes and isoscapes in geospatial forensic investigations. Isoscapes: Understanding movement, pattern, and process on Earth through isotope mapping. Dordrecht: Springer Netherlands; 2010. pp. 357–387. doi:10.1007/978-90-481-3354-3_17

64. Meier-Augenstein W. Stable Isotope Forensics: An Introduction to the Forensic Application of Stable Isotope Analysis. Stable Isotope Forensics: An Introduction to the Forensic Application of Stable Isotope Analysis. 2010. doi:10.1002/9780470688762

65. Bartelink EJ, Berg GE, Chesson LA, Tipple BJ, Beasley MM, Prince-Buitenhuys JR, et al. Applications of stable isotope forensics for geolocating unidentified human remains from past conflict situations and large-scale humanitarian efforts. New Perspectives in Forensic Human Skeletal Identification. 2017. doi:10.1016/B978-0-12-805429-1.00015-6

Database_S1

Click here to access/download
Supporting Information
Database_S1.csv

Click here to access/download
Supporting Information
Database_S2.csv

Database_S3

Click here to access/download
Supporting Information
Database_S3.csv

Click here to access/download
Supporting Information
Data_S1.csv

Data_S2

Click here to access/download
Supporting Information
Data_S2.csv

Click here to access/download
Supporting Information
Script_S1.R

Script_S2

Click here to access/download
Supporting Information
Script_S2.R
