# Supplementary material for: Multi-isotopes in human hair: A tool to initiate cross-border collaboration in international cold-cases
Source: PLoS One. 2022 Oct 26;17(10):e0275902. doi: 10.1371/journal.pone.0275902 (PMC9603990; doi:10.1371/journal.pone.0275902)
Supplement: Attachment — Submitted filename: Response_to_Reviewers.DOCX [file pone.0275902.s009.DOCX]

PONE-D-22-13353

Multi-isotopes in Human Hair: A tool to initiate Cross-border Collaboration in International Cold-Cases

PLOS ONE

Dear Dr. Bataille,

Thank you for submitting your manuscript to PLOS ONE. After careful consideration, we feel that it has merit but does not fully meet PLOS ONE’s publication criteria as it currently stands. Therefore, we invite you to submit a revised version of the manuscript that addresses the points raised during the review process.

Both reviewers underlined the quality and interest of the submitted manuscript. Reviewer 2 raises a relevant point on the use of international and/or in-house standards to verify the validity of inter-laboratory comparisons. This needs to be added to the material and method section. Information on the time range recorded by the hair in general (in comparison to other tissues) and the sampling pattern used for the study in particular is pertinent and should be also added. Additional minor corrections are listed in both reviews.

Please submit your revised manuscript by Jul 22 2022 11:59PM. If you will need more time than this to complete your revisions, please reply to this message or contact the journal office at [plosone@plos.org](mailto:plosone@plos.org). When you're ready to submit your revision, log on to <https://www.editorialmanager.com/pone/> and select the 'Submissions Needing Revision' folder to locate your manuscript file.

Please include the following items when submitting your revised manuscript:

- A rebuttal letter that responds to each point raised by the academic editor and reviewer(s). You should upload this letter as a separate file labeled 'Response to Reviewers'.
- A marked-up copy of your manuscript that highlights changes made to the original version. You should upload this as a separate file labeled 'Revised Manuscript with Track Changes'.
- An unmarked version of your revised paper without tracked changes. You should upload this as a separate file labeled 'Manuscript'.

If you would like to make changes to your financial disclosure, please include your updated statement in your cover letter. Guidelines for resubmitting your figure files are available below the reviewer comments at the end of this letter.

If applicable, we recommend that you deposit your laboratory protocols in protocols.io to enhance the reproducibility of your results. Protocols.io assigns your protocol its own identifier (DOI) so that it can be cited independently in the future. For instructions see: <https://journals.plos.org/plosone/s/submission-guidelines#loc-laboratory-protocols>. Additionally, PLOS ONE offers an option for publishing peer-reviewed Lab Protocol articles, which describe protocols hosted on protocols.io. Read more information on sharing protocols at <https://plos.org/protocols?utm_medium=editorial-email&utm_source=authorletters&utm_campaign=protocols>.

We look forward to receiving your revised manuscript.

Kind regards,

Dorothée Drucker
Academic Editor
PLOS ONE

Journal Requirements:

When submitting your revision, we need you to address these additional requirements.

1. Please ensure that your manuscript meets PLOS ONE's style requirements, including those for file naming. The PLOS ONE style templates can be found at 
<https://journals.plos.org/plosone/s/file?id=wjVg/PLOSOne_formatting_sample_main_body.pdf> and 
<https://journals.plos.org/plosone/s/file?id=ba62/PLOSOne_formatting_sample_title_authors_affiliations.pdf>

We have ensured that our manuscript followed all the PLOS ONE’s style requirements including for file naming

2. Please provide additional details regarding participant consent. In the ethics statement in the Methods and online submission information, please ensure that you have specified what type you obtained (for instance, written or verbal, and if verbal, how it was documented and witnessed). If your study included minors, state whether you obtained consent from parents or guardians. If the need for consent was waived by the ethics committee, please include this information.

We have provided this information by adding the “informed written consent”. Our study did not include minors.

3. Thank you for stating the following in the Acknowledgments Section of your manuscript:

"This project was supported by the Chemical, Biological, Radiological-Nuclear and
Explosives Research and Technology Initiative [Award No. CRTI08-0116RD] awarded to
G.S.J., the Canadian Security and Safety Program Targeted Investment [CSSP-2018-TI2385] awarded to C.P.B. This research was further supported by the American Academy of
Forensic Sciences (AAFS) Humanitarian and Human Rights Resource Center (HHRRC)
grant, supported by the AAFS and National Institute of Justice (U.S. Department of Justice),
awarded to S.T.M.A. Work by G.J.B. was supported by US National Science Foundation
grants DBI-1565128 and DBI-1759730. The authors would like to thank the anonymous hair
donors for supporting this research. The authors would also like to thank Paul Middlestead
and the U of Ottawa's Jan Veizer Stable Isotope Laboratory for assistance in stable isotope
analysis. "

We note that you have provided funding information that is not currently declared in your Funding Statement. However, funding information should not appear in the Acknowledgments section or other areas of your manuscript. We will only publish funding information present in the Funding Statement section of the online submission form. 
Please remove any funding-related text from the manuscript and let us know how you would like to update your Funding Statement. Currently, your Funding Statement reads as follows:

"This project was supported by the Chemical, Biological, Radiological-Nuclear and Explosives Research and Technology Initiative [Award No. CRTI08-0116RD] awarded to G.S.J., the Canadian Security and Safety Program Targeted Investment [CSSP-2018-TI-2385] awarded to C.P.B. This research was further supported by the American Academy of Forensic Sciences (AAFS) Humanitarian and Human Rights Resource Center (HHRRC) grant, supported by the AAFS and National Institute of Justice (U.S. Department of Justice), awarded to S.T.M.A. Work by G.J.B. was supported by US National Science Foundation grants DBI-1565128 and DBI-1759730. "

Please include your amended statements within your cover letter; we will change the online submission form on your behalf.

We have removed the funding information from the acknowledgments. The funding information above are correct and contain all the information. We have added it to the cover letter.

4. We note that Figures 1, 2, 4, and 6 in your submission contain [map/satellite] images which may be copyrighted. All PLOS content is published under the Creative Commons Attribution License (CC BY 4.0), which means that the manuscript, images, and Supporting Information files will be freely available online, and any third party is permitted to access, download, copy, distribute, and use these materials in any way, even commercially, with proper attribution. For these reasons, we cannot publish previously copyrighted maps or satellite images created using proprietary data, such as Google software (Google Maps, Street View, and Earth). For more information, see our copyright guidelines: <http://journals.plos.org/plosone/s/licenses-and-copyright>.

This is incorrect. None of the figures contain any proprietary information. All the figures were designed under CC License. The images are not satellite images they are probability maps generated by the R script given in supplementary material. All the images can be published under the CC BY 4-0. The country polygons on the maps are from Natural Earth. We added a mention: “Coastlines and country boundaries are from <http://www.naturalearthdata.com/>” in Figs 1,2, 4 and 6.
 
We require you to either (1) present written permission from the copyright holder to publish these figures specifically under the CC BY 4.0 license, or (2) remove the figures from your submission:
 
a. You may seek permission from the original copyright holder of Figures 1, 2, 4, and 6 to publish the content specifically under the CC BY 4.0 license.  
 
We recommend that you contact the original copyright holder with the Content Permission Form (<http://journals.plos.org/plosone/s/file?id=7c09/content-permission-form.pdf>) and the following text:
“I request permission for the open-access journal PLOS ONE to publish XXX under the Creative Commons Attribution License (CCAL) CC BY 4.0 (<http://creativecommons.org/licenses/by/4.0/>). Please be aware that this license allows unrestricted use and distribution, even commercially, by third parties. Please reply and provide explicit written permission to publish XXX under a CC BY license and complete the attached form.”
 
Please upload the completed Content Permission Form or other proof of granted permissions as an ""Other"" file with your submission.
 
In the figure caption of the copyrighted figure, please include the following text: “Reprinted from [ref] under a CC BY license, with permission from [name of publisher], original copyright [original copyright year].”
 
b. If you are unable to obtain permission from the original copyright holder to publish these figures under the CC BY 4.0 license or if the copyright holder’s requirements are incompatible with the CC BY 4.0 license, please either i) remove the figure or ii) supply a replacement figure that complies with the CC BY 4.0 license. Please check copyright information on all replacement figures and update the figure caption with source information. If applicable, please specify in the figure caption text when a figure is similar but not identical to the original image and is therefore for illustrative purposes only.
The following resources for replacing copyrighted map figures may be helpful:
 
USGS National Map Viewer (public domain): <http://viewer.nationalmap.gov/viewer/>
The Gateway to Astronaut Photography of Earth (public domain): <http://eol.jsc.nasa.gov/sseop/clickmap/>
Maps at the CIA (public domain): <https://www.cia.gov/library/publications/the-world-factbook/index.html> and <https://www.cia.gov/library/publications/cia-maps-publications/index.html>
NASA Earth Observatory (public domain): <http://earthobservatory.nasa.gov/>
Landsat: <http://landsat.visibleearth.nasa.gov/>
USGS EROS (Earth Resources Observatory and Science (EROS) Center) (public domain): [http://eros.usgs.gov/#](http://eros.usgs.gov/)
Natural Earth (public domain): <http://www.naturalearthdata.com/>

5. Please include captions for your Supporting Information files at the end of your manuscript, and update any in-text citations to match accordingly. Please see our Supporting Information guidelines for more information: <http://journals.plos.org/plosone/s/supporting-information>.

We have modified the file naming and added the requested captions to the supporting information.

6. Please review your reference list to ensure that it is complete and correct. If you have cited papers that have been retracted, please include the rationale for doing so in the manuscript text, or remove these references and replace them with relevant current references. Any changes to the reference list should be mentioned in the rebuttal letter that accompanies your revised manuscript. If you need to cite a retracted article, indicate the article’s retracted status in the References list and also include a citation and full reference for the retraction notice.

We have verified the reference list.

[Note: HTML markup is below. Please do not edit.]

Reviewers' comments:

Reviewer's Responses to Questions

**Comments to the Author**

1. Is the manuscript technically sound, and do the data support the conclusions?

The manuscript must describe a technically sound piece of scientific research with data that supports the conclusions. Experiments must have been conducted rigorously, with appropriate controls, replication, and sample sizes. The conclusions must be drawn appropriately based on the data presented.

Reviewer #1: Yes

Reviewer #2: Yes

2. Has the statistical analysis been performed appropriately and rigorously?

Reviewer #1: I Don't Know

Reviewer #2: Yes

3. Have the authors made all data underlying the findings in their manuscript fully available?

The [PLOS Data policy](http://www.plosone.org/static/policies.action#sharing) requires authors to make all data underlying the findings described in their manuscript fully available without restriction, with rare exception (please refer to the Data Availability Statement in the manuscript PDF file). The data should be provided as part of the manuscript or its supporting information, or deposited to a public repository. For example, in addition to summary statistics, the data points behind means, medians and variance measures should be available. If there are restrictions on publicly sharing data—e.g. participant privacy or use of data from a third party—those must be specified.

Reviewer #1: Yes

Reviewer #2: Yes

4. Is the manuscript presented in an intelligible fashion and written in standard English?

PLOS ONE does not copyedit accepted manuscripts, so the language in submitted articles must be clear, correct, and unambiguous. Any typographical or grammatical errors should be corrected at revision, so please note any specific errors here.

Reviewer #1: Yes

Reviewer #2: Yes

5. Review Comments to the Author

Please use the space provided to explain your answers to the questions above. You may also include additional comments for the author, including concerns about dual publication, research ethics, or publication ethics. (Please upload your review as an attachment if it exceeds 20,000 characters)

Reviewer #1: The present manuscript demonstrates the importance of international cooperation in elucidating the identity of unknown dead. The isoscapes produced illustrate the regional differences in the isotopic values of sulphur and hydrogen in hair samples from the North American population and are an important tool for getting closer to the region of origin of UBCs and other unknown individuals. In principle, however, when creating isoscapes it must be ensured that the analytical data of all database samples are comparable within the limits of measurement uncertainty. In the isotopic analysis of human hair, calibration of all database samples against the reference values of the currently available internationally recognised hair standards (USGS-42 and USGS-43) is essential.
It is not clear from the manuscript whether the isotopic values of all hair samples were calibrated against the USGS-42 and USGS-43 reference values before the isoscapes were created. Does this also apply to the sulphur isotope data of the Mexican hair samples taken from Ammer et al. 2020, because the original literature does not contain any precise information on this? Indeed, inter-laboratory comparisons show that the sulphur isotope data in hair in different laboratories can differ by several ‰ if no official or internal hair standards are taken into account as reference values.

We thank the author for this excellent and critical comment. We have rewritten most of the method isotope section to detail the traceability of δ^2^H and δ^34^S isotope data. We added some information to demonstrate that all samples are traceable to the same scale. For δ^34^S, all results are traceable to the Vienna Canyon Diablo Triolite (VCDT) scale via IAEA-S-1, IAEA-S-2 and IAEA-S-3. We wanted to point to the reviewer that USGS42 and USGS43 are themselves calibrated to IAEA-S-1, IAEA-S-2 and IAEA-S-3. Data from Mexico were analyzed for *δ*^34^S values at UC Davis Stable Isotope Facility, which used 6 internal RMs including keratin calibrated against IAEA-S-1, IAEA-S-2 and IAEA-S-3. Data from the USA was obtained from the SIRFER Laboratory at the University of Utah and were calibrated to 3 internal RMs calibrated using IAEA-S-1, IAEA-S-2 and IAEA-S-3. As a quality check, we asked SIRFER to rerun new USGS42 and USGS43 using the same analytical protocol and the obtained values for USGS42 (7.94±0.06‰ (n=5)) and USGS43 (10.37±0.13‰ (n=5)) compare well with the expected values for USGS42 (7.84 ± 0.25 ‰) and USGS43 (10.46 ± 0.22 ‰). Again, as USGS42 and USGS43 are themselves calibrated against IAEA-S-1, IAEA-S-2 and IAEA-S-3, this analysis technically superfluous. Data from Canada (and the forensic cases) was obtained from the Veizer Stable Isotope Lab at the University of Ottawa. RMs used for calibration were IAEA-S-1 (−0.3 ‰), IAEA-S-2 (22.7 ‰) and IAEA-S-3 (−32.6 ‰). The values used for IAEA-S-2 and IAEA-S-3 were not the same as used in the other laboratories. To verify these measurement are compatible with the other measurements, both USGS42 and USGS43 were analyzed against IAEA-S-1, IAEA-S-2 and IAEA-S-3, and the measured *δ*^34^S values and one standard deviation (7.58 ± 0.13 ‰ (n=3; USGS42) and 10.22 ± 0.15 ‰ (n=3; USGS43)) overlapped with the certified δ^34^S values and uncertainties for USGS42 and USGS43.RMs used for calibration were directly IAEA-S-1, IAEA-S-2 and IAEA-S-3 making them directly comparable to other dataset. Again to validate the approach, we analyzed USGS42 and USGS43 as QC and obtained measured values that overlapped with the certified *δ*^34^S values and uncertainties for these standards.

For *δ*^2^H values, data from Mexico were analyzed at the Jan Veizer Stable Isotope Laboratory at the University of Ottawa using the comparative analysis approach. However, instead of using only CBS and KHS for calibration, a three-point calibration with CBS, KHS and USGS43 was used for generating these data. This three-point calibration has technically a slightly different equation as the two-point calibration using CBS and KHS only. However, the effect on the obtained *δ*^2^H values is minimal (<0.7‰) and negligible relative to analytical uncertainty. Additionally, USGS42 and USGS43 values were re-calibrated using the CBS and KHS comparative approach (Soto et al. 2017) and their values fall within analytical uncertainty of the certified values (Coplen et al. 2016). Consequently, USGS42 and USGS43 are also traceable to the VSMOW-SLAP scale. Finally, USGS42 was included as a quality check in all hair analysis sequences and the measured values were within the certified values and uncertainties. The *δ*^2^H data from the USA and Canada were analyzed using older protocols and calibration standards, and were recalibrated in a previous study (Magozzi et al. 2021). This study provides a high level of details on traceability. After uncertainty propagation the rescaled *δ*^2^H values have relatively large uncertainty ~3‰. Based on these facts, the *δ*^2^H data generated and compiled in this study are all traceable to the Vienna Standard Mean Ocean Water-Standard Light Antarctic Precipitation (VSMOW-SLAP) scale.

For the regional classification of the individuals, isotope signature of their hair samples were used. The bulk analysis of hair from the UBCs yields mean values that contain information on the food and beverages consumed during the last 4 months of life. During this period of life, the individuals probably no longer stayed in their home region, but were "travelling" and exposed to more or less random food. These mean values are thus hardly compatible with the individuals' regions of origin, and may also feign stays in regions where they have never been. Ultimately, the informative value of the hair is limited to the time before death, and with the bulk analysis of a hair sample of 4 cm, it is not possible to differentiate the whereabouts (or changes of diet, health problems or starvation) during the last weeks of life, as for example with UBC#2.
Segmental analyses of the hair samples might have improved the regional allocations. A more precise regional assignment could have been expected from the examination of bones or teeth of the individuals.

We agree with this comment and we have added some sentences in the methods and in the discussion to underline that the bulk hair analyses is not the ideal approach.

Isotopic examination of these body tissues of UBCs should be the next step to get closer to their identity with the help of the isoscapes created in this work.

Yes this has actually been done isotopes were analyzed in bones and teeth as well (see Saskia Ammer’s doctoral thesis) but we limited this paper to hair as we did not develop the bones and teeth isoscapes. We added a sentence to underline this point.

My special comments on the manuscript are in the PDF version.

We followed most of the recommendations of the reviewer in the pdf version including adding references, changing figure 4 and adding some small clarifications throughout the manuscript. The only comment we did not follow is to modify the hair growth rate to 1.4 cm per month. While this is true that 1.4 cm per month might be a better approximation of the growth rate, the age model of this individual is pretty elastic considering that we used a dreadlock and had difficulty aligning hair (this was mentioned in the method section). We added this detail in the figure caption: “Due to the difficulty in aligning the hair of Mr. Halifax, the timeframe represented by hair length is more elastic than usual [10,35]. We used an approximate hair growth rate of 1cm per month for visualization.”

Reviewer #2: Overall, this is an excellent paper and makes good use of existing reference data to test out on real unidentified remains cases, including a case study from Canada (where the decedent likely spent time in the US) and case studies of deceased undocumented border crossers. This paper is in excellent shape and only needs minor edits. It will make a great contribution to PLOS One and to forensic isotope literature.

Minor comments and edits:
Page 3, line 20: change "compose" to "are assimilated into keratin"
We modified as suggested.

Page 3, line 25: change "it's hair inherits" to "their hair incorporates"
We modified as suggested.

Page 3, line 31: change "inherited" to "assimilated"
We modified as suggested.

Page 3: line 34: add values after delta-34S
We added as suggested.

Page 3, line 36: change "also composing" to "are also incorporating"
We modified as suggested.

Page 4, line 4: change "in average" to "on average"
We modified as suggested.

Page 4, line 5: soybeans are C3 plants (not C4)
We removed soybeans as suggested.

Page 5, line 7: change to "US Border Patrol"
We changed as suggested.

Page 5, line 19: change "isotope" to "isotopes"
We modified as suggested.

Page 7, lines 5-6: change "Canada demonstrating" to "Canada would demonstrate"
We modified as suggested.

Page 8, line 5: change "Americans" to "US Americans"
We modified as suggested.

Page 10, line 20: change to "with larger datasets"
We modified as suggested.

Page 10, line 21: change "approach" to "approaches"
We modified as suggested.

Page 11, line 1: change to "dual isotope probabilistic maps"
We modified as suggested.

In several places, "data is" is used and it should be "data are". Check to make sure plural phrasing is used.
We modified as suggested.

When discussing border crossers, please add the word deceased so it's clear that the samples are from decedents, not the living.
We added as suggested.

6. PLOS authors have the option to publish the peer review history of their article ([what does this mean?](https://journals.plos.org/plosone/s/editorial-and-peer-review-process#loc-peer-review-history)). If published, this will include your full peer review and any attached files.


If you choose “no”, your identity will remain anonymous but your review may still be made public.


**Do you want your identity to be public for this peer review?** For information about this choice, including consent withdrawal, please see our [Privacy Policy](https://www.plos.org/privacy-policy).

Reviewer #1: No

Reviewer #2: No

[NOTE: If reviewer comments were submitted as an attachment file, they will be attached to this email and accessible via the submission site. Please log into your account, locate the manuscript record, and check for the action link "View Attachments". If this link does not appear, there are no attachment files.]

While revising your submission, please upload your figure files to the Preflight Analysis and Conversion Engine (PACE) digital diagnostic tool, <https://pacev2.apexcovantage.com/>. PACE helps ensure that figures meet PLOS requirements. To use PACE, you must first register as a user. Registration is free. Then, login and navigate to the UPLOAD tab, where you will find detailed instructions on how to use the tool. If you encounter any issues or have any questions when using PACE, please email PLOS at [figures@plos.org](mailto:figures@plos.org). Please note that Supporting Information files do not need this step.

*In compliance with data protection regulations, you may request that we remove your personal registration details at any time.* [*(Remove my information/details)*](https://www.editorialmanager.com/pone/login.asp?a=r)*. Please contact the publication office if you have any questions.*
